# Vagus Nerve Preservation for Early Distal Gastric Cancer With Monitoring and Indocyanine Green Labeling: A Randomized Clinical Trial

**DOI:** 10.1001/jamasurg.2024.5077

**Published:** 2024-11-13

**Authors:** Zhibo Yan, Meng Wei, Tongchao Zhang, Jinghao Guo, Ao Yu, Yize Liang, Yadi Huang, Xiaohan Cui, Honglei Wang, Kuiquan Zhou, Zikun Dong, Wenbin Yu

**Affiliations:** 1 Department of Gastrointestinal Surgery, General Surgery, Qilu Hospital of Shandong University, Jinan, China; 2 Clinical Epidemiology Unit, Qilu Hospital of Shandong University, Jinan, China; 3 Clinical Research Center of Shandong University, Jinan, China; 4 Department of General Surgery, Tengzhou Central People’s Hospital, Tengzhou, China

## Abstract

**Importance:**

Radical gastric cancer surgery can cause functional and physiological disorders due to the resection of perigastric vagus nerves. Few studies have used intraoperative neurophysiological monitoring and indocyanine green (ICG) labeling to preserve the perigastric vagus nerve and to evaluate the corresponding effects.

**Objective:**

To assess the feasibility and effects of vagus nerve preservation using neurophysiologic monitoring and ICG labeling during laparoscopic distal gastrectomy in patients with early distal gastric cancer.

**Design, Setting, and Participants:**

This open-label, prospective randomized clinical trial initially enrolled 285 patients with clinical stage cT1N0M0 distal gastric cancer from May 2022 to May 2023. This trial was conducted at Qilu Hospital of Shandong University in Jinan, China, and enrolled patients aged 18 to 80 years with histologically proven gastric adenocarcinoma scheduled for distal gastrectomy. The final follow-up examination was performed May 1, 2024.

**Interventions:**

Eligible participants were randomly assigned 1:1 to vagus nerve preservation distal gastrectomy (VPG) or vagus nerve resection distal gastrectomy (VRG).

**Main Outcomes and Measures:**

The primary outcome was the incidence of postsurgical gastroparesis. Secondary outcomes included postoperative gallstone formation, quality of life, morbidity, mortality, overall survival, and disease-free survival up to 12 months postoperatively. All analyses were based on both intention-to-treat and per-protocol analyses.

**Results:**

Of 264 patients included in the intention-to-treat analysis, the median (IQR) patient age was 58.0 (52.0-67.0) years, and 67 patients (25.4%) were female. Both the VPG and VRG groups included 132 patients. Postoperative gastroparesis occurred in 1 patient (0.8%) in the VPG group and in 10 patients (7.6%) in the VRG group. Gallstones developed in 0 patients in the VPG group and in 9 patients (6.8%) in the VRG group. As assessed by mean (SD) score on the 30-item European Organization for Research and Treatment of Cancer Quality of Life Questionnaire, the VRG group experienced more nausea and vomiting at 6 months postsurgery (19.38 [7.62]) than the VPG group (17.15 [9.21]) (*P* = .03) and had significantly higher rates of persistent appetite loss, reflux symptoms, and eating difficulties at both 6 months and 12 months than the VPG group. Differences in postoperative complications and metastasis were not significant.

**Conclusions and Relevance:**

Neurophysiologic monitoring and ICG labeling during distal laparoscopic gastrectomy for vagus nerve preservation in patients with early distal gastric cancer are safe and feasible. Preserving the perigastric vagus nerve may retain the function of the remnant stomach and improve quality of life.

**Trial Registration:**

Chictr.org.cn Identifier: ChiCTR2200059489

## Key Points

**Question:**

Is vagus nerve preservation during distal gastrectomy for early gastric cancer using intraoperative neurophysiological monitoring and indocyanine green labeling feasible, and is it superior to conventional distal gastrectomy?

**Findings:**

In this randomized clinical trial of 264 patients with early-stage distal gastric cancer, vagus nerve preservation resulted in significantly less postoperative gastroparesis compared to vagus nerve resection (0.8% vs 7.6%), lower incidence of gallstone formation (0% vs 6.8%), and better quality of life.

**Meaning:**

Vagus nerve preservation using intraoperative neurophysiological monitoring and indocyanine green labeling during distal gastrectomy for early gastric cancer is safe and achieved favorable effects for patients compared with vagus nerve resection.

## Teaser

This randomized clinical trial assesses the feasibility and effects of vagus nerve preservation using neurophysiologic monitoring and indocyanine green labeling during laparoscopic distal gastrectomy in patients with early distal gastric cancer at 1 hospital in Jinan, China.

## Introduction

As a high-prevalence tumor, especially in East Asia, Eastern Europe, and South America, gastric cancer is a globally life-threatening disease.^1^ Partially due to the development of endoscopic instruments and government-guided screening programs, the number and proportion of patients with early gastric cancer (EGC) are rising, and gastric cancer–associated mortality has substantially reduced.^2^ Despite the progress that has been made in the understanding of gastric cancer biology, radical surgery with standard lymph node dissection is still the preferred method for curative intent.^3^ However, conventional gastrectomy usually results in a low postoperative quality of life (QOL) known as postgastrectomy syndrome, due to altered form and function of the stomach.^4^ Since the 5-year survival rate of EGC has reached 90%,^5^ the focus of interest is thus shifting to the functional outcome and QOL.

Among the existing functional preserving gastric surgeries, the vagus nerve preservation surgery for distal gastrectomy is attracting significant attention. Previous studies have mainly focused on the associations between hepatic branch preservation and the incidence of gallbladder stone formation after surgery.^6^ However, the preservation of the celiac branch or other perigastric branches of the vagus nerves is relatively understudied and controversies regarding its benefit remain^8^ This controversy over the benefit of vagus nerve preservation can be attributed in part to the possibility of unnoticed injuries to the vagus nerve. Due to the lack of specific markers during the surgery and the celiac branches being located relatively close to the left gastric artery, the identification and functional preservation of the vagus nerve is challenging. Moreover, the preservation of the celiac branch possibly hinders thorough lymph node dissection around the left gastric artery.^10^

Intraoperative neurophysiologic monitoring (IONM) has been applied in thyroid and spinal surgeries for decades.^11^ Previous studies have confirmed this technique in determining the de-innervation or preservation of perigastric vagus nerves during gastrectomy surgery.^13^ In addition, as a novel and noticeable intraoperative navigation technology, indocyanine green (ICG) fluorescence–guided laparoscopic gastric surgery enables better tumor localization,^14^ more accurate identification of sentinel lymph nodes (LNs), and an increased number of harvest LNs.^16^ Thus, the combination of ICG fluorescence and IONM may facilitate not only reliable nerve protection, but also accurate identification and thorough lymph node dissection. A systematic review^17^ revealed that among lymph node stations 1 to 7, those along the lesser gastric curvature (station 3) exhibited the highest rate of metastases; thus, we intraoperatively basin resected the station 3 LNs and ICG-positive station 1 LNs, followed by examination via frozen section. Patients without metastasis in the resected lymph nodes were enrolled, and a randomized clinical trial was conducted to assess the feasibility and effect of vagus nerve preservation surgery.

## Methods

### Study Design

This open-label, prospective randomized clinical trial was performed at Qilu Hospital of Shandong University in Jinan, China, from May 2022 to May 2023. The follow-up period ended on May 1, 2024. The study protocol was approved by the ethics committee of Qilu Hospital of Shandong University and is available in Supplement 1. Written informed consent was obtained from all of the participants based on sufficient acknowledgment of the trial’s procedure. A data and safety monitoring committee reviewed the data. This study followed the Consolidated Standards of Reporting Trials (CONSORT) reporting guidelines for randomized clinical trials.

### Study Population

The inclusion criteria for this study were (1) patients were 18 to 80 years old with histologically proven gastric adenocarcinoma at cT1N0M0 staging, assessed according to the eighth edition of the American Joint Committee on Cancer tumor, node, metastasis classification; (2) patients were scheduled for distal gastrectomy with D1/D1+ lymphadenectomy and possible for R0 surgery by this procedure; and (3) patients were able to tolerate general anesthesia. Patients who had undergone prior endoscopic mucosal resection (EMR) or endoscopic submucosal dissection (ESD) were considered eligible if they met the following criteria: (1) pathological examination indicated the necessity for further gastrectomy; (2) distal gastrectomy could be conducted within 3 months after EMR or ESD; and (3) no perforation or other serious complications occurred during the preceding EMR or ESD procedures. Patients were excluded if they met the following criteria: (1) allergic to iodine or specific contrast agents; (2) had recurrent gastric cancer; (3) with lymph node metastasis, distant metastasis, or direct invasion of the pancreas, spleen, or other organs nearby in the preoperative examinations; (4) had received neoadjuvant or adjuvant chemotherapy treatment; (5) suffering from other serious diseases, including cardiovascular, respiratory, kidney, or liver disease, complicated by poorly controlled hypertension, diabetes, mental disorders, or diseases; (6) need for combined organ resection due to aggression of gastric cancer of other diseases; or (7) need for concurrent surgeries due to other surgical diseases.

### Randomization

Individuals who met all inclusion criteria underwent surgery, and ICG injections were performed under gastroscopy 1 day before surgery. The randomization to receive vagus nerve preservation distal gastrectomy (VPG) or vagus nerve resection distal gastrectomy (VRG) (1:1 ratio) was performed after a full exploration of the abdomen and confirmation that no metastasis occurred in the basin dissection of station 3 LNs and ICG-positive station 1 LNs by intraoperative cryosection. The data center used SAS version 9.2 (SAS Institute) to produce sequential numbers, which were requested by the chief surgeons. These numbers ranged from 1 to 260 and corresponded to the intervention assignments. An independent masked coordinator (J.G.) performed the follow-up and the surgeons were masked to treatment assignments during all postoperative follow-up visits.

### Interventions

After randomization, patients were operated on by 2 surgeons with experience of more than 50 laparoscopic gastrectomies^18^ who are also proficient in performing mini-laparotomy and totally laparoscopic gastrectomies. In the VRG group, the resection range and lymph node dissection were followed by the Japanese Gastric Cancer Treatment Guidelines 2018 (fifth edition),^19^ while in the VPG group, the hepatic branches of the anterior vagal nerve were preserved, and ICG-negative station 1 LNs were retained to protect the anterior and posterior gastric branches of the vagus nerve. IONM was used to identify and preserve the nerve. Nasogastric tubes were routinely placed and were removed after surgery when evidence of bowel function returned. The diagrams and photographs depicting vagus nerve protection using IONM are available in eFigures 1 and 2 in Supplement 2.

### Follow-Up

The masked trial coordinator recorded all patient symptoms and outcomes every day during hospitalization. The patients were followed up for 12 months, and follow-up was achieved for all patients after surgery. The follow-up schedule was 2 weeks, 1 month, and every 3 months in the first year after surgery. Routine physical examination and laboratory tests, including blood cell count, carcinoembryonic antigen, carbohydrate antigen 19-9, cancer antigen 125, carbohydrate antigen 72-4, and alpha-fetoprotein, were performed on each visit. Chest and abdominal computed tomography (CT) scanning were performed starting from 1 month postoperatively.

### Outcome Measurements

The study’s primary end point was the incidence of postsurgical gastroparesis (PSG) within 12 months postoperation.

PSG was diagnosed using the following procedure: (1) nasogastric tube drainage volume of more than 800 mL per day or the presence of nasogastric tubes 10 days postoperatively. Then, upper gastrointestinal radiography and/or gastroscopy were performed to verify the existence of delayed stomach emptying and the absence of any physical obstructions impeding gastric outflow; (2) no apparent irregularities in the balance of fluids and electrolytes; (3) no hidden medical condition, such as hypothyroidism or choroiditis, that could be a potential cause of PSG; and (4) no ongoing medication treatment that could impact the contractile function of smooth muscles.^20^ It was hypothesized that the incidence of PSG would be decreased in the VPG group. The secondary end points were the comparison of postoperative gallstone formation, QOL, morbidity, mortality, overall survival, and disease-free survival between the 2 groups.

Scores on the European Organization for Research and Treatment of Cancer Quality of Life (EORTC QLQ) 30-item core questionnaire (EORTC QLQ-C30), version 3, and the EORTC QLQ 22-item stomach cancer–specific questionnaire (EORTC QLQ-STO22) were assessed before surgery and at 6 and 12 months after surgery.

For the EORTC QLQ-C30, these assessments included 4 items: (1) the global health status scale, an evaluation of overall health and well-being; (2) functional scales, which included 5 different scales assessing physical, role, emotional, cognitive, and social functioning; (3) symptom scales, which included 3 symptom scales to gauge the severity of fatigue, pain, nausea, and vomiting; and (4) single items, which are individual questions that cover specific issues, such as dyspnea, insomnia, appetite loss, constipation, diarrhea, and financial difficulties.

The EORTC QLQ-STO22 questionnaire was assessed separately, with a focus on the following gastric cancer–related aspects: (1) gastric cancer–related scales, which involved 5 different scales that assessed dysphagia, eating restriction, pain, reflux, and anxiety and (2) single items, which included 4 individual items that examined issues like dry mouth, body image, taste changes, and hair loss.

On the EORTC QLQ-C30, items for each domain were averaged to obtain a mean score, which was then standardized to a 0 to 100 scale per EORTC guidelines. Higher scores on functioning and global health scales indicated better health, whereas higher scores on symptom scales indicated worse symptoms.

### Sample Size

Based on a retrospective study,^20^ the incidence of PSG in laparoscopic radical gastrectomy was 6.9%, and it was assumed that the preservation of the vagus nerve would prevent the occurrence of PSG; thus, the minimum sample size to detect a difference with 80% statistical power (α = .05, 2-sided test) was 106 per group. Taking the dropout rate of 15% into account, at least 125 patients per group were needed. The sample size was calculated using nQuery Advisor version 7.0 (Statistical Solutions).

### Statistical Analysis

Data were analyzed using SPSS version 27.0 (IBM). Continuous variables were tested for normality using the Kolmogorov-Smirnov test, and variables that met normality were represented by mean and SD; otherwise, variables were represented by the median with interquartile range. Categorical variables were given as numbers and percentages. When comparing 2 groups for continuous variables, unpaired *t* tests and adjusted unpaired *t* tests were used for normally distributed data with equal variances or missing variance, while the Mann-Whitney *U* test was applied for non-normally distributed data. Categorical variables were compared using χ^2^ tests or Fisher exact tests. Both intention-to-treat (ITT) and per-protocol analyses were conducted. All statistical tests were 2-sided, and a *P* value less than .05 was considered statistically significant.

## Results

### Study Population

**Figure 1.**
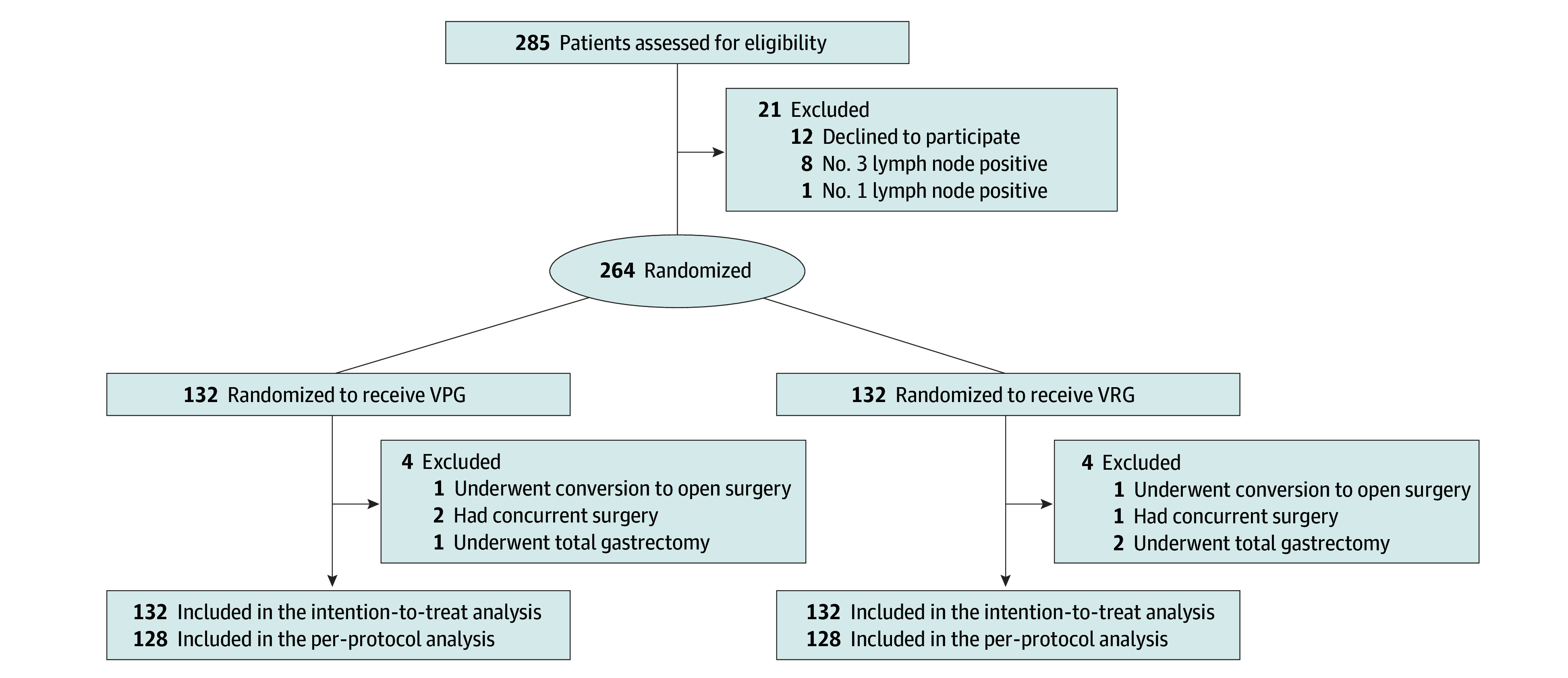
CONSORT Flow Diagram VPG indicates vagus nerve preservation distal gastrectomy; VRG, vagus nerve resection distal gastrectomy.

The study flowchart is summarized in Figure 1. From May 2022 to May 2023, 285 patients were identified as having EGC in Qilu Hospital of Shandong University in Jinan, China, and were initially identified as eligible for the study. Among these patients, 12 patients declined to participate, 9 patients had positive station 3 or station 1 LNs, and the remaining 264 patients were regarded as the ITT population and randomly assigned 1:1 to the VPG and VRG groups. Two patients underwent conversion, 3 had concurrent surgery, and 3 underwent total gastrectomy. After follow-up, 128 patients from each group were considered in the per-protocol analysis. The baseline characteristics of the patients are shown in Table 1. Median (IQR) patient age was 58.0 (52.0-67.0) years, and 67 patients (25.4%) were female. The baseline characteristics of the patients who were pathologically diagnosed as pT1N0M0 were further analyzed in eTable 1 in Supplement 2.

**Table 1.** Baseline Characteristics of Patients

Variable	Patients, No. (%)			
	Intention-to-treat analysis		Per-protocol analysis	
	VPG (n = 132)	VRG (n = 132)	VPG (n = 128)	VRG (n = 128)
Sex				
Female	32 (24.2)	35 (26.5)	31 (24.2)	34 (26.6)
Male	100 (75.8)	97 (73.5)	97 (75.8)	94 (73.4)
Age, median (IQR), y	58.5 (53.0-68.0)	58.0 (52.0-66.0)	58.0 (52.8-68.3)	58.0 (52.0-66.3)
BMI, mean (SD)^a^	24.86 (3.29)	24.88 (3.52)	24.87 (3.21)	24.90 (3.58)
Smoking				
Yes	45 (34.1)	57 (43.2)	44 (34.4)	55 (43.0)
No	87 (65.9)	75 (56.8)	84 (65.6)	73 (57.0)
Alcohol consumption				
Yes	34 (25.8)	38 (28.8)	32 (25.0)	36 (28.1)
No	98 (74.2)	94 (71.2)	96 (75.0)	92 (71.9)
ASA score				
I	31 (23.5)	41 (31.1)	30 (23.4)	41 (32.0)
II	94 (71.2)	83 (62.9)	91 (71.1)	80 (62.5)
III	7 (5.3)	8 (6.1)	7 (5.5)	7 (5.5)
Comorbidities				
Diabetes	23 (17.4)	37 (28.0)	23 (18.0)	37 (28.9)
CHD	16 (12.1)	19 (14.4)	16 (12.5)	19 (14.8)
Hypertension	27 (20.5)	32 (24.2)	27 (21.1)	32 (25.0)
Marital status				
Married	119 (90.2)	118 (89.4)	115 (89.8)	114 (89.1)
Unmarried	13 (9.8)	14 (10.6)	13 (11.2)	14 (11.9)
Education				
≤Primary	24 (18.2)	24 (18.2)	24 (18.8)	24 (18.8)
High school	56 (42.4)	55 (41.7)	54 (42.2)	53 (41.4)
University or college	52 (39.4)	53 (40.2)	50 (39.0)	51 (39.8)
Working status				
Employed	72 (54.5)	70 (53.0)	70 (54.7)	68 (53.1)
Unemployed	60 (45.5)	62 (47.0)	58 (45.3)	60 (46.9)
Tumor size, mean (SD), mm	21.97 (13.41)	20.60 (12.46)	22.1 (13.6)	20.6 (12.6)
pT stage				
T1a	69 (52.3)	76 (57.6)	67 (52.3)	74 (57.8)
T1b	55 (41.7)	54 (40.9)	53 (41.4)	52 (40.6)
T2	5 (3.8)	2 (1.5)	5 (3.9)	2 (1.6)
T3	3 (2.3)	0	3 (2.3)	0
pN stage				
N0	114 (86.4)	117 (88.6)	110 (85.9)	113 (88.3)
N1	12 (9.1)	13 (9.8)	12 (9.4)	13 (10.2)
N2	5 (3.8)	2 (1.5)	5 (3.9)	2 (1.6)
N3	1 (0.8)	0	1 (0.8)	0
Histology				
Differentiated	67 (50.8)	57 (43.2)	65 (50.8)	56 (43.8)
Undifferentiated	65 (49.2)	75 (56.8)	63 (49.2)	72 (56.3)
Anastomosis method				
Mini-laparotomy	53 (40.2)	59 (44.7)	49 (38.3)	55 (43.0)
Total laparoscopic	79 (59.8)	73 (55.3)	79 (61.7)	73 (57.0)
Reconstruction type				
Billroth I	2 (1.5)	3 (2.3)	2 (1.6)	3 (2.3)
Billroth II	129 (97.7)	127 (96.2)	126 (98.4)	125 (97.7)
Roux-en-Y	1 (0.8)	2 (1.5)	0	0
Postoperative AC				
Yes	18 (13.6)	14 (10.6)	18 (14.1)	14 (10.9)
No	114 (86.4)	118 (89.4)	110 (85.9)	114 (89.1)

Abbreviations: AC, adjuvant chemotherapy; ASA, American Society of Anesthesiologists; BMI, body mass index; CHD, coronary heart disease; VPG, vagus nerve preservation distal gastrectomy; VRG, vagus nerve resection distal gastrectomy.

^a^ Calculated as weight in kilograms divided by height in meters squared.

### Surgical Outcomes

In the ITT analyses, the mean (SD) operation time of the VPG group (196.10 [23.59] minutes) was significantly longer than that of the VRG group (179.08 [21.11] minutes). However, the estimated mean (SD) blood loss (VPG, 75.89 [50.21] mL vs VRG, 78.62 [51.81] mL) did not differ significantly. No significant differences were found regarding the postoperative complications in terms of bleeding, anastomosis leakage, and pancreatic fistula (VPG, 3.1% vs VRG, 2.4%). No surgery-related death occurred in either group. The surgical outcomes in the per-protocol analysis (Table 2) and pathologically diagnosed pT1N0M0 populations showed similar differences (eTable 2 in Supplement 2).

**Table 2.** Surgical Outcomes of Vagus Nerve Preservation (VPG) and Vagus Nerve Resection (VRG) Distal Gastrectomy

Surgical outcome	Patients, No. (%)					
	Intention-to-treat analysis			Per-protocol analysis		
	VPG (n = 132)	VRG (n = 132)	*P* value	VPG (n = 128)	VRG (n = 128)	*P* value
Operation time, mean (SD), min	196.10 (23.59)	179.08 (21.11)	<.001	195.03 (23.49)	177.72 (19.50)	<.001
Blood loss, mean (SD), mL	75.89 (50.21)	78.62 (51.81)	.66	71.89 (20.51)	74.32 (13.28)	.26
Surgeon						
A	94 (71.2)	86 (65.2)	.29	91 (71.1)	83 (64.8)	.28
B	38 (28.8)	46 (34.8)		37 (28.9)	45 (35.2)	
Postoperative complications						
Bleeding	1 (0.8)	1 (0.8)	.57	1 (0.8)	1 (0.8)	.57
Pancreatic fistula	3 (2.3)	1 (0.8)		3 (2.3)	1 (0.8)	
Anastomotic leakage	0	1 (0.8)		0	1 (0.8)	
Postoperative gastroparesis	1 (0.8)	10 (7.6)	.006	1 (0.8)	10 (7.8)	.006
Gallstone	0	9 (6.8)	.007	0	9 (7.0)	.003
Postoperative hospital stays, mean (SD), d	8.12 (2.25)	9.20 (4.04)	.02	8.10 (2.25)	9.52 (3.73)	.003
Regular diet, mean (SD), wk	6.81 (1.14)	7.59 (2.59)	.001	6.77 (1.05)	7.56 (2.60)	.002
Metastasis	0	1 (0.8)	>.99	0	1 (0.8)	>.99
Overall survival	132 (100)	132 (100)	NA	128 (100)	128 (100)	NA

Abbreviation: NA, not applicable.

### PSG

The median (IQR) follow-up duration on the last follow-up date (May 1, 2024) was 16 (12-24) months. No significant gastroparesis was observed before surgery or after postoperative adjuvant chemotherapy. After 1-year follow-up, in the VRG group, 3 patients experienced PSG within 7 days after surgery, 6 patients experienced PSG within 8 to 30 days after surgery, and 1 patient experienced PSG within 1 to 12 months after surgery. In the VPG group, 1 patient developed PSG 13 days after surgery. All patients recovered from PSG and resumed a regular diet; however, patients in the VRG group took a longer mean (SD) time to recovery (7.59 [2.59] weeks) than those in the VPG group (6.81 [1.14] weeks; *P* = .001). Data on the duration of PSG are shown in eTable 3 in Supplement 2.

### Gallstone Formation

No gallstones were found on CT scanning before surgery, and no stones were detected in the bile duct or intrahepatic duct throughout the study period. Zero patients in the VPG group and 9 of 132 patients in the VRG group (6.8%) developed gallstones within 12 months after gastrectomy. Data regarding the proportions of patients who developed gallstones at different time points can be found in eTable 4 in Supplement 2.

### QOL

**Figure 2.**
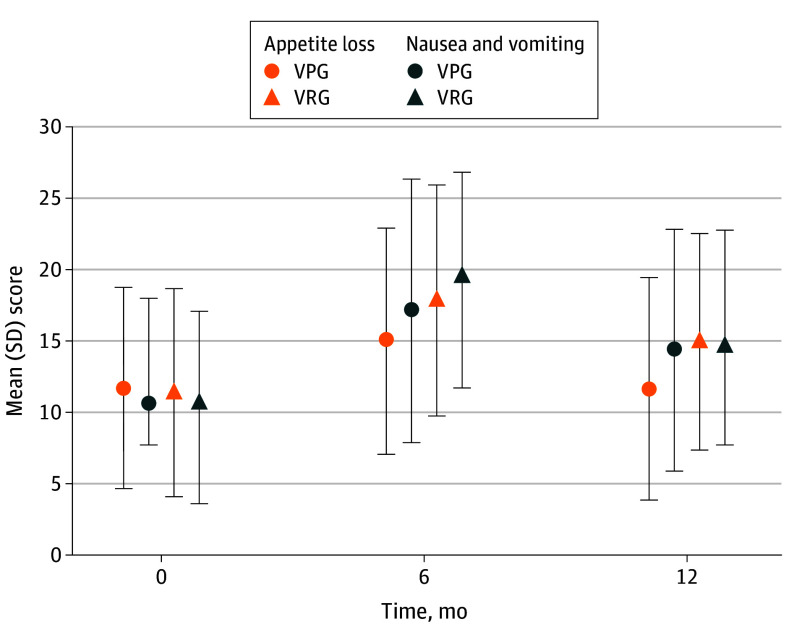
The Appetite Loss and Nausea and Vomiting Scales of the European Organization for Research and Treatment of Cancer Quality of Life 30-Item Core Questionnaire (EORTC QLQ-C30) VPG indicates vagus nerve preservation distal gastrectomy; VRG, vagus nerve resection distal gastrectomy.

**Figure 3.**
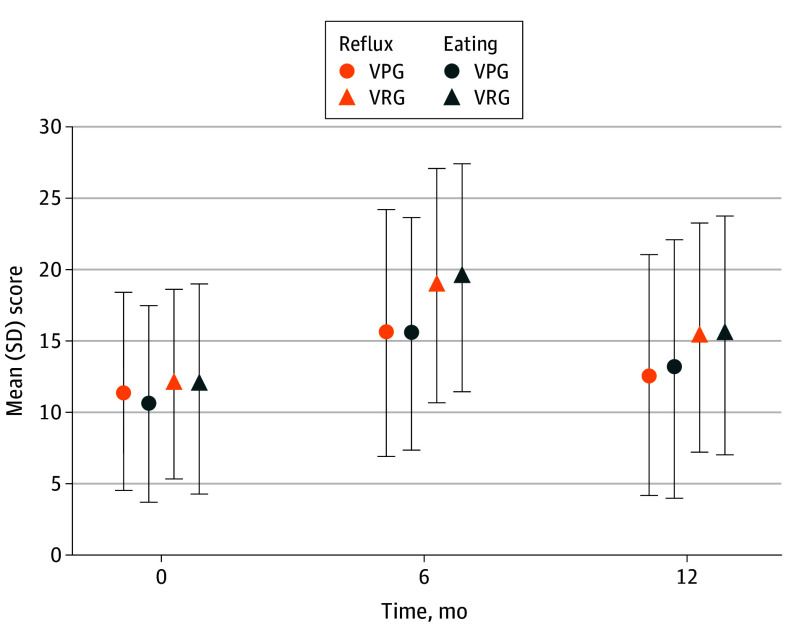
The Reflux and Eating Scales of the European Organization for Research and Treatment of Cancer Quality of Life 22-Item Stomach Cancer–Specific Questionnaire (EORTC QLQ-STO22) VPG indicates vagus nerve preservation distal gastrectomy; VRG, vagus nerve resection distal gastrectomy.

No significant difference was observed in the preoperative QOL scores. Regarding the mean (SD) EORTC QLQ-C30 scores, the VRG group showed significant appetite loss symptoms (VRG, 17.87[8.10] vs VPG, 15.02 [7.92]; *P* = .004) and nausea and vomiting symptoms (VRG, 19.38 [7.62] vs VPG, 17.15 [9.21]; *P* = .03) at 6 months after surgery. Nausea and vomiting changed overtime, with no difference observed between the two groups at 12 months postoperation. However, a significant appetite loss was observed in the VRG group (14.97 [7.56] vs VPG, 11.66 [7.77]; *P* = .001; Figure 2). No statistical difference was found between the 2 groups regarding the functioning scales, fatigue, pain, dysponea, insomnia, constipation, diarrhea, and financial difficulties (eFigures 3-5 in Supplement 2). Regarding gastric cancer-specific QOL in eFigures 6 and 7 in Supplement 2, no obvious difference were found between the 2 groups concerning dysphagia, pain, anxiety, dry mouth, taste, body image, and hair loss. However, in the VRG group, higher reflux symptoms (VRG, 18.90 [8.19] vs VPG, 15.57 [8.63]; *P* = .001), and eating symptoms (VRG, 19.42 [8.01] vs VPG, 15.51 [8.17]; *P* < .001) were observed 6 months after surgery. Though the symptoms were alleviated in both groups over time, persistent higher reflux symptoms (VRG, 15.27 [8.07] vs VPG, 12.59 [8.42]; *P* = .009) and eating symptoms (VRG, 15.45 [8.40] vs VPG, 13.07 [9.09]; *P* = .03) existed in the VRG group compared with the VPG group 12 months postoperation (Figure 3). Per-protocol analysis showed similar differences as the ITT analysis (eTables 5 and 6 in Supplement 2). The populations with or without postoperative chemotherapy are analyzed in eTables 7 and 8 in Supplement 2.

## Discussion

Vagus nerve-preservation gastrectomy has garnered considerable interest in recent years.^22^ Studies suggest that hepatic branch preservation reduces postoperative gallstone formation.^7^ However, these studies were limited by their retrospective nature and small sample sizes. The benefits of celiac branch preservation remain controversial,^9^ with some speculating that successful vagus nerve preservation during the procedure could influence outcomes. Additionally, safeguarding the vagus nerve while performing a thorough lymph node dissection increases the surgical complexity.

To reduce the surgical difficulty and ensure the consistency of the surgical techniques, IONM and ICG labeling were used during the surgery. To the best of our knowledge, this is the first study to investigate the role of preserving the anterior and posterior gastric branches of vagus nerve during distal gastrectomy.

PSG is known to worsen pain, delay adjuvant chemotherapy, and increase the risk of tumor recurrence and metastasis.^25^ As a multifactorial disease, PSG is influenced by various factors, with perigastric vagus nerve preservation being just 1 contributing element.^26^ To assess the impact of vagus nerve preservation on PSG, excluding other factors, patients with ECG without severe preoperative comorbidities were selected as the study population. Consistent with previous studies,^27^ PSG incidence was 7.6% in the VRG group and decreased to 0.8% with VPG.

Another common complication after gastrectomy is gallstone formation.^29^ A meta-analysis^23^ found that gallstone occurred in 296 of 1558 patients (19.0%) after distal gastrectomy. Meanwhile, 64.7% of the gallstones were detected within 1 year postgastrectomy.^29^ In this study, gallstone incidence was 0% in the VPG group and 6.8% in the VRG group. Consistent with the meta-analysis,^23^ resection of the hepatic branch of the vagus nerve was strongly and consistently related to gallstone formation.

Concerning the QLQ-C30, a decline in the overall QOL was observed 6 months postoperatively, irrespective of the preservation of the vagus nerve. Both groups showed gradual improvement over time; however, the VPG group had better scores in appetite loss, nausea, and vomiting symptoms. These improvements may relate to remnant gastric fundus motility and ghrelin secretion, both regulated by the perigastric vagus nerve.^30^ For QLQ-STO22, the persistent higher reflux symptoms in VRG group imply a possibility of reflux esophagitis occurring 6 and 12 months after surgery.

Regarding the safety of the procedure, IONM was safely applied to identify perigastric nerves.^13^ The study team has extensive experience with ICG fluorescence-guided laparoscopic gastrectomy.^32^ Combining these techniques is feasible and safe, and the procedure showed no inferiority compared with conventional laparoscopic surgery. One patient showed hepatic metastasis in VRG, while no recurrence or metastasis occurred in the VPG group after 1-year follow-up. Moreover, station 3 LNs have a much higher metastasis rate compared with station 1 LNs, and no metastasis in station 3 LNs and ICG-positive station 1 LNs is the premise of preserving the ICG-negative station 1 LNs.^33^

### Limitations

Several limitations should be noted in our study. First, all patients were from 1 single Chinese high-volume gastric cancer center, and whether this technique can be generalized to other institutions or other ethnicities should be further validated. Second, the follow-up time was relatively short. However, PSG usually occurs within 1 month postoperation. In addition, 64.7% of the gallstones were detected within 1 year postgastrectomy.^29^ Thus, a minimum 1-year follow-up period is sufficient for exploring the main study end points. An extended follow-up period will be performed to evaluate the effects on the tumor prognosis. Third, while consistent with previous studies, PSG diagnosis primarily relies on symptoms and upper gastrointestinal radiography, as no universally accepted criterion standard exists. Fourth, the diagnosis of gallstones primarily relies on CT examinations, with a lower positivity rate compared to ultrasound or magnetic resonance imaging.

## Conclusions

These results showed that delicately preserving the perigastric vagus nerve by using both IONM and ICG labeling significantly reduced the incidence of PSG and gallstone formation and improved patient QOL within a 1-year follow-up period. Studies involving a larger and more diverse patient population, with longer follow-up periods, are needed to explore the safety and feasibility of this technique in the future.

## Notes

Supplement 1. Trial Protocol

Supplement 2. **eTable 1.** Baseline Characteristics of Patients Pathlogically Diagnosed as pT1N0M0 **eTable 2.** Surgical Outcomes of Patients Pathlogically Diagnosed as pT1N0M0 **eTable 3.** Information on the Duration of PSG After Gastrectomy in Intention-to-Treat Analysis **eTable 4.** The Proportion of Patients Developing Gallstones Within 3, 6, 9, and 12 Months After Gastrectomy in Intention-to-Treat Analysis **eTable 5.** Quality of Life and Functional Outcome of Patients Followed by VPG and VRG Before Surgery, and 6, and 12 Months After Surgery in Intention-to-Treat Analysis **eTable 6.** Quality of Life and Functional Outcome of Patients Followed by VPG and VRG Before Surgery, and 6, and 12 Months After Surgery in Per-Protocol Analysis **eTable 7.** Quality of Life and Functional Outcomes in Patients Receiving Postoperative Adjuvant Chemotherapy Followed by VPG and VRG Before Surgery, and 6, and 12 Months After Surgery in Intention-to-Treat Analysis **eTable 8.** Quality of Life and Functional Outcomes in Patients Not Receiving Postoperative Adjuvant Chemotherapy Followed by VPG and VRG Before Surgery, and 6, and 12 Months After Surgery in Intention-to-Treat Analysis **eFigure 1.** Diagram For Perigastric Vagus Nerve and the Preservation of Vagus Nerve During Distal Gastrectomy **eFigure 2.** The Preservation of the Vagus Nerve During Distal Gastrectomy **eFigure 3.** The Functional Scales of the EORTC QLQ-C30 **eFigure 4.** The Fatigue, Pain, Dysponea, and Insomnia Scales of the EORTC QLQ-C30 **eFigure 5.** The Constipation, Diarrhea, and Financial Difficulties Scales of the EORTC QLQ-C30 **eFigure 6.** The Dysphagia, Pain, and Anxiety Scales of the EORTC QLQ-STO22 **eFigure 7.** The Dry Mouth, Taste, Body Image, and Hair Loss Scales of the EORTC QLQ-STO22

Supplement 3. Data Sharing Statement
