# Supplementary material for: Vagus Nerve Preservation for Early Distal Gastric Cancer With Monitoring and Indocyanine Green Labeling: A Randomized Clinical Trial
Source: JAMA Surg. 2024 Nov 13:e245077. Online ahead of print. doi: 10.1001/jamasurg.2024.5077 (PMC11561724; doi:10.1001/jamasurg.2024.5077)
Supplement: Supplement 1. — Trial Protocol [file jamasurg-e245077-s001.pdf]

Vagus Nerve Preservation for Early Distal Gastric Cancer Using Neurophysiological Monitoring and Indocyanine Green Labeling

Study Protocol

Research center: Qilu Hospital of Shandong University

Principle investigator:

Wenbin Yu, MD, PhD

Department of Gastrointestinal Surgery, General Surgery, Qilu Hospital, Shandong University.

107 Wenhuaxi Road, Jinan, 250012, China.

Telephone: +86-531-82166331

E-mail: wenbin_yu2003@163.com

Summary

Protocol Title	Vagus Nerve Preservation for Early Distal Gastric Cancer Using Neurophysiological Monitoring and Indocyanine Green Labeling
Protocol Version	Version: 1.5
PI	Wenbin Yu
Research centers	Qilu Hospital of Shandong University
Indications	Patients with early gastric cancer are scheduled to undergo curative laparoscopic gastric resection.
Research purpose	To assess the feasibility and effect of vagus nerve preservation by combined application of intraoperative neurophysiologic monitoring and indocyanine green during laparoscopic distal gastrectomy in patients with early-stage gastric cancer.
Research Design	Prospective, single-center, open-label, randomized controlled trial.
Case Grouping	Group A (Study Group): Laparoscopic distal gastrectomy with preservation of vagus nerve<br>Group B (Control Group): Laparoscopic distal gastrectomy without preservation of vagus nerve
Determination of Sample Size	Based on a retrospective study, the incidence of postsurgical gastroparesis syndrome in laparoscopic radical gastrectomy was 6.9%, and we assumed that the preservation of the vagus nerve would prevent the occurrence of PSG, thus the minimum sample size to detect a difference with 80% statistical power (α = .05; 2-sided test) was 106 per group. Taking the dropout rate of 15% into account, at least 125 patients for each group are needed. The sample size was calculated by using nQuery Advisor version 7.0 (Statistical Solutions).
Inclusion Criteria	(1) Patients were 18-80 years old with histologically proven gastric adenocarcinoma at cT1N0M0 staging as assessed according to the

	8th edition of the American Joint Committee on Cancer (AJCC) TNM classification. (2) Scheduled for distal gastrectomy with D1/D1+ lymphadenectomy, and possible for R0 surgery by this procedure. (3) Able to tolerate general anesthesia. Patients who had undergone prior EMR or ESD were considered eligible if they met the following criteria: (1) Pathological examination indicated the necessity for further gastrectomy. (2) Distal gastrectomy could be conducted within 3 months after EMR or ESD. (3) No perforation or other serious complications occurred during the preceding EMR or ESD procedures.
Exclusion Criteria	(1) Allergic to iodine or specific contrast agents; (2) Recurrent gastric cancer; (3) With lymph node metastasis or distant metastasis, direct invasion of the pancreas, spleen, or other organs nearby in the preoperative examinations; (4) Patients who received neoadjuvant or adjuvant chemotherapy treatment; (5) Suffering from other serious diseases, including cardiovascular, respiratory, kidney, or liver disease, complicated by poorly controlled hypertension, diabetes, mental disorders or diseases; (6) Need for combined organ resection due to aggression of gastric cancer of other diseases; (7) Need for concurrent surgeries due to other surgical diseases.
Withdraw Criteria	1. The patient requested to withdraw or suspend treatment for personal reasons at any stage after the patient enrolled in the study.<br>2. The treatment implemented is proven to violate study protocol.<br>3. Simultaneous surgical treatment of other diseases.<br>4. Patients intraoperatively confirmed have to undergo total gastrectomy.<br>5. All cases of conversion to open surgery will be withdrawn.
Intervention	For patients who are assigned to the VPG group, the hepatic branch, anterior and posterior gastric branches that innervation of the remnant

	stomach were identified by intraoperative neurophysiologic monitoring and preserved.
Endpoints	Primary Outcome Measures was the incidence of postsurgical gastroparesis (PSG) within postoperative 12 months.<br>The secondary endpoints were the comparison of postoperative gallstone formation, gastric cancer specific QoL, morbidity, mortality, overall survival, and disease-free survival between the 2 groups.
Statistical Considerations	Both intention-to-treat (ITT) and per-protocol analyses were conducted.<br>Kolmogorov-Smirnov test, Student's unpaired *t*-test, adjusted Student's unpaired *t*-test, Mann-Whitney U test, and Chi-square test will be used in this study.<br>All statistical tests were two-sided, and a *P* value of <0.05 was considered statistically significant.

Table of contents

1. Background
2. Purpose
3. Study design
   3.1 Single center
   3.2 Case group
   3.3 Estimate sample size
   3.4 Blind method
   3.5 Research cycle
4. Study Objects
   4.1 Inclusion criteria
   4.2 Exclusion criteria
   4.3 Withdrawal criteria
   4.4 Selection of subjects
      4.4.1 General condition Initial evaluation
      4.4.2 Tumor-specific evaluation
5. Safety evaluation criteria, including side effects, and evaluation and reporting methods
   5.1 Variables measured
   5.2 Side effects
      5.2.1 Serious Adverse Events (SAE)
      5.2.2 Adverse Event Reporting
6. End Point
   6.1 Primary Outcome Measures
      6.1.1 The incidence of postsurgical gastroparesis
   6.2 Secondary Outcome Measures
      6.2.1 Postoperative gallstone formation
      6.2.2 Gastric cancer specific QoL
7. Diagnostic Criteria for This Study
   7.1 The AJCC-8th TNM tumor staging system will be used for this study
   7.2 Diagnostic criteria and classification of gastric cancer

7.3 Definition of early stage
7.4 Definition of distal gastric cancer
7.5 Definition of No. 3 lymph nodes
7.6 Definition of hepatic branches of the anterior vagal trunk
7.7 Definition of anterior/posterior gastric branches of the vagal trunk
8. Qualifications of The Participating Surgeons
9. Standard Operating Procedures (SOPs)
9.1 Case Selection
9.1.1 Selection of Assessment Items
9.1.2 Selection Application
9.2 Preoperative Management
9.3 Endoscopic injection of ICG
9.4 Standardization of Surgical Procedures
9.4.1 Principle of Surgical Treatments for both group
9.4.2 Observation Items During Surgery
9.5 Postoperative Management
9.5.1 Preventive Use of Analgesics
9.5.2 Fluid Replacement and Nutritional Support
9.5.3 Postoperative Rehabilitation Management
9.5.4 Patient Discharge Standards
9.5.5 Postoperative Observation Items
9.5.6 Visit plan and schedule
9.6 Benefits and risks to the subjects
10. Statistical Analysis
10.1 Definition of the Population Set for Statistical Analysis
10.2 Analysis of primary efficacy endpoints
10.3 Analysis of secondary efficacy endpoints
11. Measures for subject safety and protection
11.1 The general plan for research ethics
11.2 Informed Consent Procedures
11.3 Subject compensation

11.4 Personal information protection plan

11.5 Additional safeguards for protecting vulnerable populations if included

12. Storage and disposal of human biological materials

1. Background

Gastric cancer is a significant global health issue, with varying incidence rates worldwide. According to the World Health Organization (WHO), gastric cancer is the fifth most common cancer globally and the third leading cause of cancer-related deaths, accounting for approximately 769,000 deaths in 2020.[1] The incidence rates of gastric cancer are higher in Eastern Asia, Eastern Europe, and South America, while lower rates are observed in North America, Africa, and Oceania.

The treatment of gastric cancer depends on several factors, including the stage of the cancer, the patient's overall health, and their preferences. Surgery is the primary treatment for resectable gastric cancer and may involve partial or total gastrectomy. Due to the rising rates of early gastric cancer, there is a considerable amount of ongoing research in the field of organ- and function-preserving surgery. The goal is to enhance the effectiveness of treatment while also improving the post-operative quality of life for patients.

Among the existing functional preserving gastric surgeries, the vagus nerve preservation surgery for distal gastrectomy is attracting more attention. Previous studies mainly focused on the relationship between hepatic branch preservation and the incidences of gallbladder stone formation after the surgery.[2,3] However, the preservation of the celiac branch or other extragastric branches of the vagus nerves is relatively understudied and controversies still exist.[4,5] Controversy over the benefit of vagus nerve preservation can be attributed in part to the possibility of unnoticed injuries to the vagus nerve. Due to the lack of specific markers during the surgery and the celiac branches being relatively close to the left gastric artery, the identification and functional preservation of the vagus nerve is full of challenges. The preservation of the celiac branch hinders the thorough lymph node dissection around the left gastric artery.

How to achieve thorough lymph node dissection while maximizing nerve protection has become a clinical challenge.

Intraoperative neurophysiologic monitoring for gastrectomy involves monitoring the integrity and function of the nervous system during surgery. This monitoring helps

surgeons identify and avoid damage to critical nerves that control various functions, such as movement or sensation, which can be at risk during the procedure.[6] Besides, a novel approach using indocyanine green (ICG) and near-infrared (NIR) fluorescence imaging has been studied in gastric cancer surgery as a promising method to improve localization of the tumor, detection of sentinel lymph nodes (SLN) and real-time lymphatic mapping.[7]

This study combines intraoperative nerve monitoring with indocyanine green navigation in an attempt to resolve the conflict between nerve protection and lymph node dissection, aiming to provide evidence for the necessity and feasibility of vagus nerve protection in radical distal gastrectomy and to offer a solution for vagus nerve protection surgery.

2. Purpose

Through prospective studies on vagus nerve preservation during radical distal gastrectomy, especially focusing on the effects of preserving the hepatic and gastric branches of the vagus nerve on both short-term and long-term patient outcomes, we aim to provide evidence supporting the necessity and feasibility of vagus nerve preservation in radical distal gastrectomy and to offer solutions for vagus nerve protection surgery.

3. Study Design

Prospective, single-center, open, parallel assignment, randomized controlled

3.1 Single center

Department of Gastrointestinal Surgery, General Surgery, Qilu Hospital, Shandong University.

3.2 Case group

Group A (Study Group): Laparoscopic distal gastrectomy with preservation of vagus nerve

Group B (Control Group): Laparoscopic distal gastrectomy without preservation of vagus nerve

3.3 Estimate sample size

Based on a retrospective study, the incidence of postsurgical gastroparesis syndrome in laparoscopic radical gastrectomy was 6.9%, and we assumed that the preservation of the vagus nerve would prevent the occurrence of PSG, thus the minimum sample size to detect a difference with 80% statistical power (α = 0.05; 2-sided test) was 106 per group. Taking the dropout rate of 15% into account, at least 125 patients for each group are needed. The sample size was calculated by using nQuery Advisor version 7.0 (Statistical Solutions).

3.4 Blind method

This research adopts an open-label design

3.5 Research cycle

1) Estimated enrollment cycle: complete enrollment within 12 months.
2) Follow-up period: begins at the enrollment of the first case and ends 12 months after the enrollment of the last case.
3) Estimated time: 2022.05-2023.05 (to complete enrollment) - 2024.05 (to complete follow-up)

4. Study objects

After initial selection, the patients who met the inclusion criteria and did not conform to the exclusion criteria were scheduled for the study. During the surgery, No.3 lymph nodes and ICG-positive No.1 lymph nodes were resected for intra-operative frozen section, and the patients without metastasis of No. 3 and No.1 lymph nodes were randomized for the study.

4.1 Inclusion criteria

(1) Patients were 18-80 years old with histologically proven gastric adenocarcinoma at cT1N0M0 staging as assessed according to the 8th edition of the American Joint Committee on Cancer (AJCC) TNM classification. (2) Scheduled for distal gastrectomy

with D1/D1+ lymphadenectomy, and possible for R0 surgery by this procedure. (3) Able to tolerate general anesthesia.

Patients who had undergone prior EMR or ESD were considered eligible if they met the following criteria: (1) Pathological examination indicated the necessity for further gastrectomy. (2) Distal gastrectomy could be conducted within 3 months after EMR or ESD. (3) No perforation or other serious complications occurred during the preceding EMR or ESD procedures.

4.2 Exclusion Criteria

(1) Allergic to iodine or specific contrast agents; (2) Recurrent gastric cancer (3) With lymph node metastasis or distant metastasis, direct invasion of pancreas, spleen, or other organs nearby in the preoperative examinations (4) Patients who received neoadjuvant or AC treatment (5) Suffering from other serious diseases, including cardiovascular, respiratory, kidney, or liver disease, complicated by poorly controlled hypertension, diabetes, mental disorders or diseases (6) Need for combined organ resection due to aggression of gastric cancer of other disease (7) Need for concurrent surgeries due to other surgical diseases.

4.3 Withdraw Criteria

1) The patient requested to withdraw or suspend treatment for personal reasons at any stage after the patient enrolled in the study.
2) The treatment implemented is proven to violate study protocol.
3) Simultaneous surgical treatment of other diseases.
4) Patients intraoperatively confirmed have to undergo total gastrectomy.
5) All cases of conversion to open surgery will be withdrawn.

4.4 Selection of subjects

4.4.1 General condition Initial evaluation:

1) Patients aged over 18 and under 80 who are admitted to the hospital for elective surgery;
2) Preoperative ECOG performance score of 0 or 1;
3) Not pregnant or lactating women;

4) No serious mental illness;
5) No history of abdominal surgery;
6) No other malignant disease history within five years;
7) No history of unstable angina or myocardial infarction within six months;
8) No history of sustained systemic corticosteroid therapy within one month;
9) No requirement for simultaneous surgical treatment of other diseases;
10) Pulmonary function test with FEV1 ≥50% of the expected value;
11) No history of cerebral infarction or cerebral hemorrhage within six months;
12) ASA score of I-III.

4.4.2 Tumor-specific evaluation:

The patients undergo the endoscopic examination and CT scanning preoperatively. The position of the tumor is located at the lower one-third of the stomach and the histopathological biopsy reveals gastric adenocarcinoma, including papillary adenocarcinoma [pap], tubular adenocarcinoma [tub], mucinous adenocarcinoma [muc], signet ring cell carcinoma [sig], and poorly differentiated adenocarcinoma [por]. In CT scanning, no indication of local invasion or distant metastasis, or no enlarged lymph nodes (with a maximum diameter ≥ 1 cm) was observed.

According to the 8th edition of the American Joint Committee on Cancer (AJCC) TNM classification, the tumor was limited to the mucosa and submucosa which are T1, N0, and M0.

5. Safety evaluation criteria, including side effects, and evaluation and reporting methods

5.1 Variables measured

To evaluate the safety of VPG, VRG, and the combined application of intraoperative neurophysiological monitoring and indocyanine green labeling, the following variables will be measured.

(1) Early complications within 30 days of surgery will be compared between the patients who received VPG and those who received VRG.

(2) Postoperative bowel recovery, diet initiation, and length of hospital stay of the patients.

(3) Surgical outcomes (intra- and post-operative complications).

5.2 Side effects

5.2.1 Serious Adverse Events (SAE)

(1) All deaths within 90 days of surgery

- Must be reported as an SAE whether or not it has a causal relationship with the surgery

(2) Permanent or significant disability that has an evident causal relationship to surgery regardless of study phase (including permanent brain lesions)

(3) When the following treatments are given due to problems determined to have a causal relationship to early postoperative complications (causal relationship with early complications will be classified as definite, probable, and possible for reporting)

- Reoperation at the present or other institution due to early complications after surgery
- Unplanned treatment in the intensive care unit of the present or other institution due to early complications after surgery

* Reoperation: surgery in an operating room requiring general anesthesia

5.2.2 Adverse Event Reporting

(1) In case of an adverse event, the treating doctor or institution's coordinator who becomes aware of the event must report to the principal investigator. If the principal investigator is unreachable, the treating doctor or institution's coordinator must act on behalf of the principal investigator and carry out his/her responsibilities.

(2) Document all relevant details of the SAE, including the date of onset, description of the event, severity, relationship to the procedure, actions taken, and outcome.

(3) Report SAEs to the Institutional Review Board (IRB) within 24 hours.

6. End Point

6.1 Primary Outcome Measures

6.1.1 The incidence of postsurgical gastroparesis

Postsurgical gastroparesis was diagnosed using the following procedure: (1) Nasogastric tube drainage volume of more than 800 mL per day or the presence of

nasogastric tubes 10 days postoperatively. Then, upper gastrointestinal radiography and/or gastroscopy were performed to verify the existence of delayed stomach emptying and the absence of any physical obstructions impeding gastric outflow; (2) no apparent irregularities in the balance of fluids and electrolytes; (3) no hidden medical condition, such as hypothyroidism or choroiditis, that could be a potential cause of PSG; (4) no ongoing medication treatment that could impact the contractile function of smooth muscles.

6.2 Secondary Outcome Measures

6.2.1 Postoperative gallstone formation

The diagnosis of gallstone formation is based primarily on the results of the abdominal CT scan results. Both abdominal ultrasound and abdominal CT should be considered in the case of bile duct gallstones or intrahepatic bile duct gallstones.

The investigator will document in the case record form (CRF) the presence of gallstones, the presence of biliary sludge, and the location of gallstones (gallbladder, common bile duct, intrahepatic bile duct), size and number of gallstones.

6.2.2 Gastric cancer specific QoL

EORTC QLQ-C30 and QLQ- STO 22 questionnaires will be used to assess changes in patients' quality of life before surgery and at 6, and 12, months after surgery.

7. Diagnostic Criteria for This Study

7.1 The AJCC-8th TNM tumor staging system will be used for this study.

7.2 Diagnostic criteria and classification of gastric cancer

According to the histopathological international diagnostic criteria, classification will be divided into papillary adenocarcinoma (pap), tubular adenocarcinoma (tub), mucinous adenocarcinoma (muc), signet ring cell carcinoma (sig), and poorly differentiated adenocarcinoma (por).

7.3 Definition of early stage

Early gastric cancer (EGC) is an invasive carcinoma involving only the stomach mucosa or submucosa, regardless of lymph node status (T1, any N). In our study, we primarily selected patients classified as cT1N0M0.

7.4 Definition of distal gastric cancer

Distal gastric cancer refers to cancer that occurs in the lower part of the stomach, specifically in the region known as the distal stomach. The stomach is anatomically divided into three portions, the upper (U), middle (M), and lower (L) parts. Distal gastric cancer includes tumors located in the lower part of the stomach, including the antrum-pylorus, the lower third of the stomach corpus, and the angle.

7.5 Definition of No. 3 lymph nodes

Perigastric lymph nodes at the lesser curvature, located along the inferior (descending) branch of the left gastric artery and the right gastric artery distal to the first gastric branch.

7.6 Definition of hepatic branches of the anterior vagal trunk

The hepatic branches of anterior vagal trunk are branches of the anterior vagal trunk that provide parasympathetic innervation to the liver and gallbladder. Each anterior vagal trunk (it may be doubled or tripled) issues 1-2 hepatic branches which pass through the superior part of the omentum minus to reach and join the hepatic (nervous) plexus before proceeding to the porta hepatis. The anterior vagal trunk is the main source of parasympathetic afferents for the hepatic plexus.

7.7 Definition of anterior/posterior gastric branches of the vagal trunk

1) The anterior gastric branches of the anterior vagal trunk are branches of the anterior vagal trunk which supply the stomach.

One long branch of it runs from the lesser curvature or parallel to it in the lesser omentum as far as the pyloric antrum to fan out into branches in a way like the digits of a crow's foot to supply the pyloric antrum and the anterior wall of the pyloric canal.

2) The posterior gastric branches of the posterior vagal trunk are branches of the posterior vagal trunk which supply the stomach.

Posterior gastric branches supply the posterior surface of the stomach and its terminal branches are known as "crow's foot" which supply the pyloric antrum and the posterior wall of the pyloric canal.

8. Qualifications of the participating Surgeons

All candidate surgeons in our study met the following criteria:

Performed at least 50 LADG, and who are also proficient in performing mini-laparotomy and totally laparoscopic gastrectomies.

Pass the blind surgical video examination.

9. Standard Operating Procedures (SOPs)

9.1 Case Selection

9.1.1 Selection of Assessment Items

The baseline data for patients from hospital admission to enrollment in the study should include the following:

1) General health status: ECOG score, height, and weight.
2) Peripheral blood parameters: Hemoglobin (Hb), red blood cell count (RBC), white blood cell count (WBC), lymphocyte count (LYM), neutrophils (NEU), neutrophil percentage (NEU%), platelet count (PLT), and monocytes (MONO).
3) Blood chemistry profile: Albumin, prealbumin, total bilirubin, indirect bilirubin, direct bilirubin, AST, ALT, cholesterol, creatinine, urea nitrogen, fasting glucose, potassium (K), calcium (Ca), chloride (Cl), sodium (Na), and CRP.
4) Serum tumor markers: CEA, CA19-9, CA72-4, CA12-5, and AFP.
5) Abdominal CT scan (slice thickness ≤ 10 mm).
6) Upper gastrointestinal endoscopic ultrasonography (EUS) with biopsy, or standard upper gastrointestinal endoscopy with biopsy if EUS is not feasible.

7) Chest X-ray (AP and lateral views) to assess cardiopulmonary conditions.
8) Resting 12-lead electrocardiogram (ECG).
9) Respiratory function tests: Forced expiratory volume in one second (FEV1) and forced vital capacity (FVC).

9.1.2 Selection Application

For eligible cases meeting all inclusion criteria and none of the exclusion criteria, engage in discussions with patients and their families and obtain signed informed consent. The application and confirmation of eligibility should be completed before surgery.

The investigator must thoroughly explain the study's purpose and contents to individuals interested in participating. The subject should acknowledge and confirm their understanding of the study details, then sign the informed consent form manually. Informed consent must be obtained before conducting any study-related procedures. Upon receiving the subject's informed consent, the investigator will assign a screening number based on the order of participation in the study.

9.2 Preoperative Management

After confirming eligibility, surgery should be performed within 10 days. If the patient's clinical condition deteriorates between selection and the scheduled surgery date, the decision to proceed with the planned elective surgery should be based on the chief surgeon's judgment. If emergency surgery is required, the case should be excluded from the per-protocol (PP) set.

For patients with nutritional risks, assess the patient's nutritional status using tools such as Nutritional Risk Screening (NRS). Prescribing oral nutritional supplements if the patient is at risk of malnutrition. In cases of severe malnutrition, consider enteral or parenteral nutrition to improve nutritional status. For elderly patients, pay attention to the assessment of elderly frailty, identify high-risk groups, and take timely intervention measures. Provide counseling and support for smoking cessation before surgery for patients with a history of smoking.

Diabetes management involves optimizing glycemic control before surgery to reduce the risk of postoperative complications. Cardiovascular risk management includes

addressing cardiovascular risk factors, such as hypertension and dyslipidemia. Thromboembolic risk management entails considering thromboprophylaxis for high-risk patients to reduce the risk of thromboembolic events. Regarding prophylactic antibiotics, it is important to administer them within one hour before surgery, with redosing as needed based on the duration of the procedure.

9.3 Endoscopic injection of ICG

One day before surgery. As a fluorescent developer, ICG (Dandong Yichuang Pharmaceutical Co., Ltd) was dissolved into a 0.625 mg/ml solutions in sterile water 1 mL of the prepared solution, containing 0.625mg of ICG was injected along the submucosa of the stomach at four points around the primary tumor, respectively, for a total volume of 4ml (a total 2.5mg ICG) (Figure. 1).

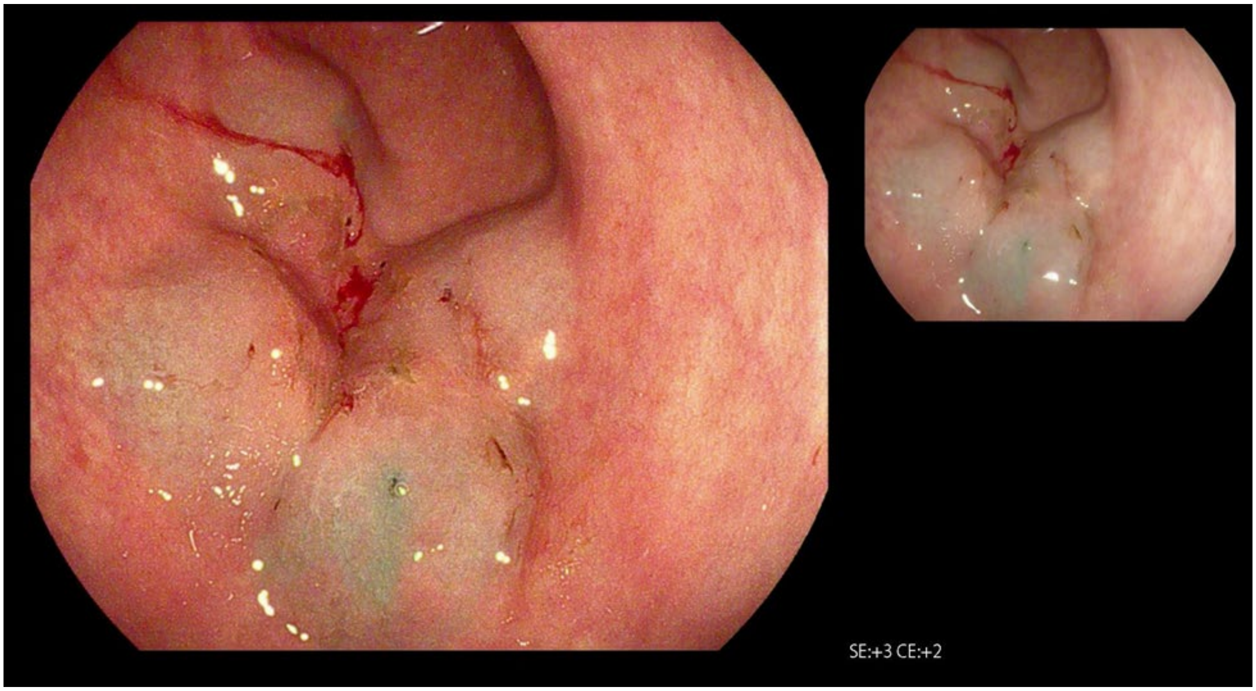

Figure. 1

9.4 Standardization of Surgical Procedures

9.4.1 Principle of Surgical Treatments for both group

9.4.1.1 Anesthesia

Surgery for laparoscopic gastric cancer is typically performed with endotracheal intubation under general anesthesia. The decision to use epidural-assisted anesthesia is at the discretion of the anesthetist and is not specified or regulated in this study.

9.4.1.2 Intraoperative Exploration

The abdominal cavity should be explored for the presence of any peritoneal, hepatic, pelvic, or mesenteric metastases, as well as for gastric serosal invasion. This exploration is crucial for accurately staging the extent of the gastric cancer and informing the treatment plan.

9.4.1.3 Gastrectomy Regulations

Follow the Japanese Gastric Cancer Treatment Guidelines 2018 (5th edition) to perform distal gastrectomy under the premise of satisfying the oncological principles.

In general, stomach resection includes the pylorus. The cardia is preserved. In the standard gastrectomy, two-thirds of the stomach is resected.

9.4.1.4 Regulations of lymph node Resection

This study protocol requires basin resection of station 3 lymph nodes and biopsy of ICG-positive station 1 lymph nodes first. In the VRG group, the resection of other lymph nodes follows the principles of D1+ radical surgery. In the VPG group, ICG-negative station 1 lymph nodes are preserved to protect the anterior and posterior gastric branches of the vagus nerve.

9.4.1.5 Regulations of perigastric nerve preservation

During the surgery, efforts were made to avoid direct contact between the active blade of the ultrasonic device and the nerve whenever possible. The hepatogastric ligament was opened to explore the subhepatic area, aiming to identify and expose the hepatic branches of the vagus nerve. Careful identification of the area between the right crus and the lesser curvature of the stomach was performed, with preservation of the anterior and posterior gastric branches of the vagus nerve.

For nerve stimulation, two stimulating probes (APS stimulator; Medtronic, Minneapolis, MN, USA) were inserted through one of the 12 mm trocars and positioned to grasp isolated nerve branches. Two 27-gauge needle electrodes (Medtronic) were

inserted through another 12 mm trocar and placed at the muscular layer of the gastric wall to detect subsequent peristaltic movement of the gastric.

9.4.1.6 Regulations of Digestive Tract Reconstruction

The method of digestive tract reconstruction is determined by the surgeon based on their experience and the specific intraoperative circumstances. If instrumental anastomosis is chosen, the surgeon decides whether manual reinforced stitching of the anastomotic stoma is necessary; the study protocol does not provide specific instructions in this regard.

9.4.1.7 Regulations of Surgery-related Equipment and Instruments

The choice of energy equipment, vascular ligation method, digestive tract cutting closure, and digestive tract reconstruction instruments is left to the discretion of the operating surgeon, who will base their decisions on their experience and the specific needs of the surgery. These details are not specified in this study protocol.

9.4.1.8 Regulations of Gastric Canal and Peritoneal Drainage Tube

Whether to leave a gastric canal or peritoneal drainage tube after surgery is decided by the surgeon based on their experience and the specific needs of the patient. This aspect is not specified in this study protocol.

9.4.1.9 Regulations of Concurrent Surgical Treatments

If a patient has another organ/system disease, the responsible surgeon and relevant department consultants will collaborate to decide if a concurrent operation is necessary and feasible. The order of operations will be determined based on clinical routine. However, such cases will be excluded from the per-protocol (PP) set according to the Exclusion Criteria.

9.4.1.10 Regulations of Photo/Image Naming and Privacy Protection

(1) All image data must be processed to ensure that no personal information of patients is disclosed.

(2) When viewing or reviewing photos/images, personal information must be obscured or processed with mosaics to protect patient confidentiality.

9.4.1.11 Basis for Confirming the Quality of Surgery

To confirm the appropriateness of the surgical procedure, surgery quality, and specimen integrity will be assessed using the photographs saved.

9.4.1.12 Storage of Image Data

All photographs and data will be saved in the hard disk or portable digital carrier in digital form, and the surgical video requires a specific hard drive to be saved for at least 3 years.

9.4.2 Observation Items During Surgery

The research assistant is responsible for documenting the relevant information on the day of surgery.

The specific items include the following:

(1) The name of the chief surgeon; (2) The duration of the operation in minutes; (3) The type of operation, extent of lymph node dissection, reconstruction method, and any intraoperative injuries; (4) Whether the operation may need to be converted to a laparotomy and the reasons for this potential change. (5) Estimation of intraoperative blood loss (ml). (6) Blood transfusion (ml). (7) Tumor location, position, and tumor size (maximum diameter, mm). (8) Tumor invasion depth, the total number of dissected lymph nodes, the number of dissected lymph nodes, and the presence of distant metastasis (location). (9) Length of proximal margin (mm), length of distal margin (mm), and radical degree of operation (R0/R1/R2). (10) Intraoperative complications: Any complications occurring from skin cutting to completion of skin stitching. (11) Intraoperative death (occurring during the period from skin cutting to completion of skin stitching) for any reason.

9.5 Postoperative Management

9.5.1 Preventive Use of Analgesics

Continuous postoperative prophylactic intravenous analgesia is generally admitted within the first 48 hours after surgery. The dose, type, and infusion rate should be determined by the anesthesiologist based on clinical practices and the patient's specific condition. Repeated use of prophylactic analgesics beyond 48 hours after surgery is not allowed unless deemed necessary.

9.5.2 Fluid Replacement and Nutritional Support

Postoperative fluid infusion (including glucose, insulin, electrolytes, vitamins, etc.) or nutritional support (enteral/parenteral) will be administered based on the doctor's

experience and routine clinical practice; no specific regulations are set in this study. After oral feeding, it is permissible to discontinue or gradually reduce fluid infusion/nutritional support.

9.5.3 Postoperative Rehabilitation Management

Management methods for incisions, stomach, and abdominal drainage tubes should follow standard diagnosis and treatment protocols. Similarly, the recovery time for eating and strategies for transitioning the diet should also adhere to regular diagnosis and treatment approaches.

9.5.4 Patient Discharge Standards

Patients are eligible for discharge if they meet the following criteria:

1) They have demonstrated satisfactory intake of a soft diet for two meals.
2) They possess limited self-care abilities.
3) They exhibit no complications upon routine clinical examinations.

The discharge event must be recorded in the Case Report Form (CRF).

9.5.5 Postoperative Observation Items

From the first day after surgery until the patient is discharged from the hospital, the research assistant should promptly complete the following tasks.

9.5.5.1 Pathological Results

1) Original lesion tissue typing: (papillary adenocarcinoma [pap], tubular adenocarcinoma [tub], mucinous adenocarcinoma [muc], signet ring cell carcinoma [sig], and poorly differentiated adenocarcinoma [por])
2) Tumor position and invasion depth
3) Histological grading (G1/G2/G3/G4/Gx)
4) Number of dissected lymph nodes and number of positive lymph nodes.

9.5.5.2 Postoperative complications

Postoperative complications are categorized into early postoperative complications and late postoperative complications. The timeframes for these categories are defined as follows:

1) Early Stage: Within 30 days after surgery, or if the postoperative hospital stay is longer than 30 days, it is considered the time of first hospital discharge.

2) Late Stage: From 30 days after surgery onward, or after the first discharge (if the postoperative stay was longer than 30 days) up to 3 years after surgery.

9.5.5.3 Upper gastrointestinal tract radiography

Upper gastrointestinal tract radiography with water-soluble contrast as a liquid suspension will be conducted at 1, 6, and 12 months post-surgery to assess food passage efficiency and detect any delayed gastric emptying in the remaining stomach. Besides, if there is a suspicion of delayed gastric emptying, upper gastrointestinal tract radiography should be performed based on the patient's condition.

9.5.5.4 Quality of life

EORTC QLQ-C30 and QLQ- STO 22 and Gastrointestinal Quality of Life Index (GIQLI) questionnaires will be used to assess changes in patients' quality of life before surgery and at 6, and 12 months after surgery.

9.5.6 Visit plan and schedule

Table I. Study flow chart sheet

Procedure	Screening /Baseline	Surgery	Postoperation to Discharge	Postoperative Day 30	Postoperative months 1~ 12	Study completion/early termination
Visit Day		0			Postoperative months 3,6,9,12	
Visit window				±7 days	±15 days	
Informed consent	Y					
Inclusion and exclusion criteria verification	Y					
Medical history, ASA, ECOG	Y					
BMI	Y			Y	Y	
Complete blood cell count	Y			Y	Y	
Biochemistry Blood Test	Y			Y	Y	
Liver and kidney function tests	Y			Y	Y	
Blood coagulation test	Y			Y	Y	
Tumor marker test[1]	Y			Y	Y	
CT scanning	Y			Y	Y	

Upper gastrointestinal tract radiography	Y		Y	Y	Y	
Upper endoscopy	Y				Y[2]	
Randomization		Y				
Recovery evaluation after surgery			Y			
Early Stage complications			Y			
Late Stage complications			Y	Y		
Pathology result			Y			
EORTC-C30, STO22 [3]	Y				Y	
Recurrence and survival					Y	Y
Case conclusion						Y

1. Tumor marker test: CEA, CA199, CA125, CA724 and AFP
2. Upper endoscopy is to be performed before surgery and at 12 months after surgery
3. EORTC-C30, STO22 questionnaires are only to be completed before surgery and at 6 and 12 months after surgery

9.6 Benefits and risks to the subjects

While the preservation of the vagus nerve during gastrectomy is not a new technique, the use of intraoperative neurophysiological monitoring or indocyanine green labeling has been deemed safe and feasible. This study represents the first to combine both techniques, which are not expected to carry high risks. Previous studies have demonstrated the potential benefits of vagus nerve preservation during gastrectomy, and successful preservation of the perigastric vagus nerves may offer advantages to patients.

10. Statistical Analysis

10.1 Definition of the Population Set for Statistical Analysis

(1) Intention-to-treat Population (ITTP): Individuals who were randomized and underwent either VPG or VRG and for whom there is at least one valid efficacy evaluation record after the intervention. The main analytical results are in line with those of the ITTP analysis.

(2) Per-protocol Population (PPP): Individuals who adhered to the study protocol, demonstrated good compliance, completed the Case Report Form (CRF), and are eligible for statistical analysis of efficacy.

10.2 Analysis of primary efficacy endpoints

(1) The incidence of postsurgical gastroparesis within 12 months after surgery.
(2) A chi-squared test or Fisher's exact test will be conducted to evaluate whether the VPG group is superior to the VRG group in terms of the incidence of postsurgical gastroparesis within 12 months after surgery. All statistical analyses will be conducted at a significance level of 5% unless otherwise specified. It should be noted that since the sample size is determined based on the power required for the primary efficacy endpoints, the power for secondary endpoints cannot be assured.

10.3 Analysis of secondary efficacy endpoints

(1) Postoperative gallstone formation

A chi-squared test or Fisher's exact test will be conducted to evaluate the differences in the incidence of postoperative gallstone formation.

(2) Quality of life

Quality of life assessments using the EORTC-C30 and STO22 questionnaires will be conducted before surgery and at 6 and 12 months after surgery. The analysis will be structured as follows:

① For the EORTC-C30 questionnaire, a separate analysis will be conducted for the five functional scales (physical, role, emotional, cognitive, and social functioning), three symptom scales (fatigue, pain and nausea, and vomiting), one global health status, and six single items.

② For the EORTC-STO22 questionnaire, a separate analysis will be conducted for the five gastric cancer-related scales (dysphagia, eating restriction, pain, reflux, and anxiety) and four single items (dry mouth, body image, taste, and hair loss).

Since the data collected will be repeated measures over time, the analysis will be performed using multilevel mixed-effects linear regression models to determine if there are differences in quality of life between the VPG and VRG groups.

(3) Postoperative complications and mortality rate

A chi-square test (or Fisher's exact test) will be utilized to assess disparities in early and late postoperative complications, as well as in the 90-day mortality rate, between the two groups

(4) Analysis of oncologic outcomes

① Disease-free survival rate: The duration from randomization to either recurrence or death will be estimated using the Kaplan-Meier Method. For patients experiencing recurrence, the time from randomization to recurrence or death will be measured, while for censored patients, the time from randomization to the censoring event will be recorded. The log-rank test will be utilized to compare the disease-free survival rate between the two groups.

② Overall survival rate: The duration from randomization to death will be estimated using the Kaplan-Meier Method. For deceased patients, the time from randomization to death will be recorded, while for censored patients, the time from randomization to the censoring event will be noted. The log-rank test will be used to compare the overall survival rate between the VPG and VRG groups.

11. Measures for subject safety and protection

11.1 General plan for research ethics

We will conduct this study in strict accordance with the most recent version of the Declaration of Helsinki (2013th edition) and will obtain approval from the IRB before initiating the study. This will selectively apply to patients with early distal gastric cancer who meet the above-mentioned oncologic factors. In the event of unexpected side effects or results, patients' safety will be the priority when responding to these matters, and a reevaluation of the study will be performed.

11.2 Informed Consent Procedures

(1) The investigator will provide an explanation to and obtain consent from subjects: co-investigators or sub-investigators listed in this study protocol may also provide information and gain consent.

(2) Person providing the consent: subjects or their representative (subjects in principle).

(3) The waiting period between imparting study information and requesting informed consent: will be decided by the subject. However, the subject will be excluded from the study if he/she cannot make the decision before the surgery.

(4) Methods to minimize the possibility of coercion or unjustified effect: there will be no additional benefit of any kind related to this clinical trial or any differences in treatment. Concerning this, subject monitoring will be routinely conducted and will be immediately reported to the IRB.

(5) Language used by the investigator when providing an explanation of the study and obtaining informed consent: Chinese. Therefore, subjects who are not familiar with Chinese and cannot understand the study in its entirety will be excluded.

(6) Language understood by subjects or their representative: Chinese. Therefore, subjects who are not familiar with Chinese and cannot understand the study in its entirety will be excluded.

(7) Information and consent form provided to the subjects and their representative:

A copy of the IRB-approved informed consent form will be provided to the subjects.

11.3 Subject compensation

(1) Incentives and compensations provided to subjects: none

(2) If a surgery-related injury occurs during the trial, appropriate treatment will be provided and every effort will be made for recovery from the side effects and minimize the cost the patient needs to pay if hospitalization is required.

(3) The institute will provide necessary treatments for the recovery from side effects that arise. However, the subjects will be responsible for the cost of inpatient or outpatient

treatment for side effects from the surgery and medical expenses not related to this study.

11.4 Personal information protection plan

(1) The patient's medical record number and pathology number will be kept in separate folders for each patient under the responsibility of the principal investigator. This information will be codified so that personal information is not identifiable. In addition, study data will be saved in a password-protected file and kept in a laboratory with a lock.
(2) Study-related records must be retained for three years following the completion of the study. If records need to be retained for longer than three years for purposes such as follow-up studies, registries, or data accumulation, the investigator must report this to the IRB.

11.5 Additional safeguards for protecting vulnerable populations if included

Vulnerable subjects are excluded from the study for the protection of subjects

12. Storage and disposal of human biological materials

Not applicable

References

1. Sung H, Ferlay J, Siegel RL, et al. Global cancer statistics 2020: GLOBOCAN estimates of incidence and mortality worldwide for 36 cancers in 185 countries. *CA: a cancer journal for clinicians.* 2021;71(3):209-249.
2. Kojima K, Yamada H, Inokuchi M, Kawano T, Sugihara K. Functional evaluation after vagus nerve–sparing laparoscopically assisted distal gastrectomy. *Surgical endoscopy.* 2008;22:2003-2008.
3. Wang C-J, Kong S-H, Park J-H, et al. Preservation of hepatic branch of the vagus nerve reduces the risk of gallstone formation after gastrectomy. *Gastric Cancer.* 2021;24:232-244.
4. Ando S, Tsuji H. SURGICAL TECHNIQUE OF VAGUS NERVE-PRESERVING GASTRECTOMY WITH D2 LYMPHADENECTOMY FOR GASTRIC CANCER. *ANZ Journal of Surgery.* 2008;78(3):172-176.
5. Furukawa H, Ohashi M, Honda M, et al. Preservation of the celiac branch of the vagal nerve for pylorus-preserving gastrectomy: is it meaningful? *Gastric Cancer.* 2018;21:516-523.
6. Kong S-H, Kim SM, Kim D-G, et al. Intraoperative neurophysiologic testing of the perigastric vagus nerve branches to evaluate viability and signals along nerve pathways during gastrectomy. *Journal of Gastric Cancer.* 2019;19(1):49-61.
7. Chen Q-Y, Xie J-W, Zhong Q, et al. Safety and efficacy of indocyanine green tracer-guided lymph node dissection during laparoscopic radical gastrectomy in patients with gastric cancer: a randomized clinical trial. *JAMA surgery.* 2020;155(4):300-311.
