# Supplementary material for: Vagus Nerve Preservation for Early Distal Gastric Cancer With Monitoring and Indocyanine Green Labeling: A Randomized Clinical Trial
Source: JAMA Surg. 2024 Nov 13:e245077. Online ahead of print. doi: 10.1001/jamasurg.2024.5077 (PMC11561724; doi:10.1001/jamasurg.2024.5077)
Supplement: Supplement 2. — eTable 1. Baseline Characteristics of Patients Pathlogically Diagnosed as pT1N0M0 eTable 2. Surgical Outcomes of Patients Pathlogically Diagnosed as pT1N0M0 eTable 3. Information on the Duration of PSG After Gastrectomy in Intention-to-Treat Analysis eTable 4. The Proportion of Patients Developing Gallstones Within 3, 6, 9, and 12 Months After Gastrectomy in Intention-to-Treat Analysis eTable 5. Quality of Life and Functional Outcome of Patients Followed by VPG and VRG Before Surgery, and 6, and 12 Months After Surgery in Intention-to-Treat Analysis eTable 6. Quality of Life and Functional Outcome of Patients Followed by VPG and VRG Before Surgery, and 6, and 12 Months After Surgery in Per-Protocol Analysis eTable 7. Quality of Life and Functional Outcomes in Patients Receiving Postoperative Adjuvant Chemotherapy Followed by VPG and VRG Before Surgery, and 6, and 12 Months After Surgery in Intention-to-Treat Analysis eTable 8. Quality of Life and Functional Outcomes in Patients Not Receiving Postoperative Adjuvant Chemotherapy Followed by VPG and VRG Before Surgery, and 6, and 12 Months After Surgery in Intention-to-Treat Analysis eFigure 1. Diagram For Perigastric Vagus Nerve and the Preservation of Vagus Nerve During Distal Gastrectomy eFigure 2. The Preservation of the Vagus Nerve During Distal Gastrectomy eFigure 3. The Functional Scales of the EORTC QLQ-C30 eFigure 4. The Fatigue, Pain, Dysponea, and Insomnia Scales of the EORTC QLQ-C30 eFigure 5. The Constipation, Diarrhea, and Financial Difficulties Scales of the EORTC QLQ-C30 eFigure 6. The Dysphagia, Pain, and Anxiety Scales of the EORTC QLQ-STO22 eFigure 7. The Dry Mouth, Taste, Body Image, and Hair Loss Scales of the EORTC QLQ-STO22 [file jamasurg-e245077-s002.pdf]

Supplemental Online Content

Yu W, Yan Z, Wei M, et al. Vagus nerve preservation for early distal gastric cancer with monitoring and indocyanine green labeling: a randomized clinical trial. *JAMA Surgery*. Published online November 13, 2024. doi:10.1001/jamasurg.2024.5077

eTable 1. Baseline Characteristics of Patients Pathlogically Diagnosed as pT1N0M0

eTable 2. Surgical Outcomes of Patients Pathlogically Diagnosed as pT1N0M0

eTable 3. Information on the Duration of PSG After Gastrectomy in Intention-to-Treat Analysis

eTable 4. The Proportion of Patients Developing Gallstones Within 3, 6, 9, and 12 Months After Gastrectomy in Intention-to-Treat Analysis

eTable 5. Quality of Life and Functional Outcome of Patients Followed by VPG and VRG Before Surgery, and 6, and 12 Months After Surgery in Intention-to-Treat Analysis

eTable 6. Quality of Life and Functional Outcome of Patients Followed by VPG and VRG Before Surgery, and 6, and 12 Months After Surgery in Per-Protocol Analysis

eTable 7. Quality of Life and Functional Outcomes in Patients Receiving Postoperative Adjuvant Chemotherapy Followed by VPG and VRG Before Surgery, and 6, and 12 Months After Surgery in Intention-to-Treat Analysis

eTable 8. Quality of Life and Functional Outcomes in Patients Not Receiving Postoperative Adjuvant Chemotherapy Followed by VPG and VRG Before Surgery, and 6, and 12 Months After Surgery in Intention-to-Treat Analysis

eFigure 1. Diagram For Perigastric Vagus Nerve and the Preservation of Vagus Nerve During Distal Gastrectomy

eFigure 2. The Preservation of the Vagus Nerve During Distal Gastrectomy

eFigure 3. The Functional Scales of the EORTC QLQ-C30

eFigure 4. The Fatigue, Pain, Dysponea, and Insomnia Scales of the EORTC QLQ-C30

eFigure 5. The Constipation, Diarrhea, and Financial Difficulties Scales of the EORTC QLQ-C30

eFigure 6. The Dysphagia, Pain, and Anxiety Scales of the EORTC QLQ-STO22

eFigure 7. The Dry Mouth, Taste, Body Image, and Hair Loss Scales of the EORTC QLQ-STO22

This supplemental material has been provided by the authors to give readers additional information about their work.

© 2024 Yu W et al. *JAMA Surgery.*

eTable 1. Baseline Characteristics of Patients Pathlogically Diagnosed as pT1N0M0

Variables	Patients, No. (%)			
	Intention-to-treat analysis		Per-protocol analysis	
	VPG(N=109)	VRG(N=115)	VPG(N=105)	VRG(N=111)
Gender				
Male	81(74.3)	86(74.8)	78(74.3)	83(74.8)
Female	28(25.7)	29(25.2)	27(25.7)	28(25.2)
Age, median [IQR], years	59.0(54.0, 68.0)	58.0(52.0, 67.0)	59.0(53.0, 69.0)	58.0(52.0, 67.0)
BMI, mean(SD), kg/m2	24.97±3.32	25.00±3.54	24.97±3.37	25.02±3.60
Smoking				
Yes	74(67.9)	62(53.9)	34(32.4)	51(45.9)
No	35(32.1)	53(46.1)	71(67.6)	60(54.1)
Alcohol consumption				
Yes	81 (74.3)	79 (68.7)	26(24.8)	34(30.6)
No	28 (25.7)	36 (31.3)	79(75.2)	77(69.4)
ASA				
I	22(20.2)	34(29.6)	21(20.0)	34(30.6)
II	82(75.2)	73(63.5)	79(75.2)	70(63.1)
III	5(4.6)	8(7.0)	5(4.8)	7(6.3)
Comorbidities				
Diabetes	23(17.4)	37(28.0)	21(20.0)	32(28.8)
CHD	16(12.1)	19(14.4)	15(14.3)	19(14.4)
Hypertension	27(20.5)	32(24.2)	23(21.9)	32(28.8)
Marital status				
Married	98(89.9)	102(88.7)	94(89.5)	98(88.3)
Unmarried	11(10.1)	13(11.3)	11(10.5)	13(11.7)
Education				
Primary or less	19(17.4)	20(17.4)	19(18.1)	20(18.0)
High school	47(43.1)	48(41.7)	45(42.9)	46(41.4)
University or college	43(39.4)	47(40.9)	41(39.0)	45(40.5)
Working status				
Employed	60(55.0)	61(53.0)	58(55.2)	59(53.2)
Unemployed	49(45.0)	54(47.0)	47(44.8)	52(46.8)
Tumor size, mean(SD), mm	20.09±12.37	20.13±12.25	20.18±12.59	20.15±12.45
pT stage				
T1a	69(63.3)	76(66.1)	67(63.8)	74(66.7)
T1b	40(36.7)	39(33.9)	38(36.2)	37(33.3)
pN stage				
N0	109(100.0)	115(100.0)	105(100.0)	111(100.0)
Histology				
Differentiated	54 (49.5)	53 (46.1)	52(49.5)	52(46.8)
Undifferentiated	55 (50.5)	62 (53.9)	53(50.5)	59(53.2)

© 2024 Yu W et al. *JAMA Surgery.*

Variables	Patients, No. (%)			
	Intention-to-treat analysis		Per-protocol analysis	
	VPG(N=109)	VRG(N=115)	VPG(N=105)	VRG(N=111)
Anastomosis method				
Mini-laparotomy	47(43.1)	50(43.5)	43(41.0)	46(41.4)
Total-laparoscopic	62(56.9)	65(56.5)	62(59.0)	65(58.6)
Reconstruction type				
Billroth I	1(0.9)	3(2.6)	1(1.0)	3(2.7)
Billroth II	107(98.2)	110(95.7)	104(99.0)	108(97.3)
Roux-en-Y	1(0.9)	2(1.7)	0(0.0)	0(0.0)

© 2024 Yu W et al. *JAMA Surgery.*

eTable 2. Surgical Outcomes of Patients Pathlogically Diagnosed as pT1N0M0

Surgical Outcomes	Patients, No. (%)					
	Intention-to-treat analysis			Per-protocol analysis		
	VPG(N=109)	VRG (N=115)	*P* value	VPG(N=105)	VRG (N=111)	*P* value
Operation time, mean(SD), min	196.58±24.25	180.02±20.11	<0.001	195.93±24.18	178.48±18.17	<0.001
Blood loss, mean(SD), ml	77.05±54.57	78.73±55.33	0.82	72.18±20.92	73.77±13.32	0.50
Surgeon			0.10			0.09
A	83 (76.1)	76 (66.1)		80(76.2)	73(65.8)	
B	26 (23.9)	39 (33.9)		25(23.8)	38(34.2)	
Postoperative complications			0.56			0.56
Bleeding	1(0.8)	1(0.8)		1(1.0)	1(0.9)	
Pancreatic fistula	3(2.3)	1(0.8)		3(2.9)	1(0.9)	
Anastomotic leakage	0(0.0)	1(0.8)		0(0.0)	1(0.9)	
Postoperative gastroparesis	1(0.9)	9(7.8)	0.03	1(1.0)	9(8.1)	0.03
Gallstone	0(0.0)	7(6.1)	0.03	0(0.0)	7(6.3)	0.03
Postoperative hospital stays, mean(SD), days	8.16±2.29	9.58±3.73	<0.001	8.14±2.29	9.23±4.21	0.02
Regular diet, mean(SD), weeks	6.84±1.18	7.62±2.72	0.007	6.79±1.08	7.59±2.74	0.006
Metastasis	0(0.0)	1(0.9)	>0.99	0(0.0)	1(0.9)	>0.99
Overall survival	109(100)	115(100)	-	105(100)	111(100)	-

© 2024 Yu W et al. *JAMA Surgery.*

eTable 3. Information on the Duration of PSG After Gastrectomy in Intention-to-Treat Analysis

Group	Time of diagnosed postoperation (day)	Time of duration (days)	The time to resume regular diet postoperation (week)
VPG (N=1)	13	16	6
VRG (N=10)	5	17	12
	6	26	14
	7	20	7
	9	12	8
	11	23	16
	11	39	15
	13	9	6
	16	31	11
	25	26	12
	53	33	26

© 2024 Yu W et al. *JAMA Surgery.*

eTable 4. The Proportion of Patients Developing Gallstones Within 3, 6, 9, and 12 Months After Gastrectomy in Intention-to-Treat Analysis

**Time spans**	**VPG (n=132) No. (%)**	**VRG (n=132) No. (%)**	***P* value**
Within 3 months	0(0.0)	1(0.8)	>0.99
Within 6 months	0(0.0)	3(2.3)	0.25
Within 9 months	0(0.0)	6(4.5)	0.029
Within 12 months	0(0.0)	9(6.8)	0.003

© 2024 Yu W et al. *JAMA Surgery.*

eTable 5. Quality of Life and Functional Outcome of Patients Followed by VPG and VRG Before Surgery, and 6, and 12 Months After Surgery in Intention-to-Treat Analysis

A. EORTC-QLQ-C30

Variables	Before surgery				6 months after surgery				12 months after surgery			
	mean(SD)		Mean diff.	*P* value	mean(SD)		Mean diff.	*P* value	mean(SD)		Mean diff.	*P* value
	VPG(N=132)	VRG(N=132)			VPG(N=132)	VRG(N=132)			VPG(N=132)	VRG(N=132)		
**Function scales**												
Global health status	76.98(8.81)	78.36(10.29)	-1.38	0.241	56.23(9.27)	57.39(9.6)	-1.16	0.316	64.17(9.85)	64.27(9.35)	-0.10	0.929
Physical functioning	87.51(7.53)	87.02(8.18)	0.49	0.611	70.59(9.63)	69.83(9.4)	0.76	0.518	75.2(8.55)	75.38(9.01)	-0.18	0.872
Emotional functioning	83.68(8.49)	84.95(8.44)	-1.27	0.226	77.52(8.72)	77.11(9.54)	0.41	0.717	85.16(8.33)	83.86(8.91)	1.30	0.221
Cognitive functioning	89.45(6.71)	88.79(6.55)	0.66	0.42	85.05(9.58)	83.33(9.32)	1.72	0.141	86.68(7.3)	87.48(7.3)	-0.80	0.377
Social functioning	87.82(7.58)	86.89(7.59)	0.93	0.319	70.88(8.86)	72.2(10.33)	-1.32	0.264	79.8(8.72)	79.45(8.73)	0.35	0.746
**Symptom scales**												
Fatigue	13.5(8.1)	12.55(7.78)	0.95	0.334	22.78(9.57)	23.77(9.63)	-0.99	0.406	19.06(10.05)	17.9(8.83)	1.16	0.321
Nausea and vomiting	10.55(7.46)	10.36(6.74)	0.19	0.836	17.15(9.21)	19.38(7.62)	-2.23	0.033	14.38(8.48)	14.51(8.27)	-0.13	0.901
Pain	10.77(7)	11.3(7.19)	-0.53	0.539	12.64(7.97)	13.83(9.17)	-1.19	0.265	12.19(7.77)	12.05(7.6)	0.14	0.879
Insomnia	14.26(8.54)	15.62(8.58)	-1.36	0.197	25.05(10.23)	25.68(9.24)	-0.63	0.601	19.66(8.57)	19.43(8.96)	0.23	0.833
Appetite loss	11.7(7.02)	11.38(7.27)	0.32	0.712	15.02(7.92)	17.87(8.10)	-2.85	0.004	11.66(7.77)	14.97(7.56)	-3.31	0.001
Constipation	14.11(8.35)	13.32(8.77)	0.79	0.451	17.56(9.3)	18.45(8.65)	-0.89	0.424	15.4(8.89)	15.01(9.07)	0.39	0.722
Diarrhea	12.07(8.04)	12.5(8.27)	-0.43	0.667	23.84(9.75)	24.05(10.5)	-0.21	0.865	18.14(9.11)	18.51(8.63)	-0.37	0.739
Financial difficulties	13.73(7.87)	15.39(9.55)	-1.66	0.126	24.44(9.95)	25.67(8.93)	-1.23	0.292	28.8(10.39)	28.61(10.26)	0.19	0.877

© 2024 Yu W et al. *JAMA Surgery.*

B. EORTC-QLQ-STO22												
**Variables**	**Before surgery**				**6 months after surgery**				**12 months after surgery**			
	**mean(SD)**		**Mean diff.**	***P* value**	**mean(SD)**		**Mean diff.**	**P value**	**mean(SD)**		**Mean diff.**	***P* value**
	**VPG(N=132)**	**VRG(N=132)**			**VPG(N=132)**	**VRG(N=132)**			**VPG(N=132)**	**VRG(N=132)**		
Dysphagia	12.04(6.8)	12.45(7.48)	-0.41	0.642	21.89(10.09)	20.01(9.63)	1.88	0.122	16.58(7.84)	15.84(8.85)	0.74	0.476
Pain	10.23(6.84)	10.27(6.76)	-0.04	0.957	16.61(8.75)	16.48(8.12)	0.13	0.901	14.97(8.48)	14.36(7.15)	0.61	0.531
Reflux	11.44(6.9)	11.98(6.67)	-0.54	0.514	15.57(8.63)	18.90(8.19)	-3.33	0.001	12.59(8.42)	15.27(8.07)	-2.68	0.009
Eating	10.61(6.91)	11.66(7.35)	-1.05	0.231	15.51(8.17)	19.42(8.01)	-3.91	<0.001	13.07(9.09)	15.45(8.40)	-2.38	0.028
Anxiety	31.02(9.44)	28.78(10.94)	2.24	0.076	32.55(9.79)	32.02(10.67)	0.53	0.674	25.42(10.06)	27.69(9.96)	-2.27	0.067
Dry mouth	26.67(10.33)	24.63(9.37)	2.04	0.095	26.06(9.66)	26.74(8.87)	-0.68	0.551	20.02(9.32)	21.64(9.3)	-1.62	0.158
Taste	11.04(7.06)	9.85(7.38)	1.19	0.182	14.33(8.47)	13.58(8.92)	0.75	0.488	12.22(7.68)	12.91(8.1)	-0.69	0.479
Body image	15.23(8.92)	16.15(7.91)	-0.92	0.378	22.73(9.61)	24.09(9.95)	-1.36	0.261	18.51(7.57)	17.56(9.23)	0.95	0.363
Hair loss	34.67(10.1)	34.64(9.71)	0.03	0.985	39.51(9.94)	41.08(9.05)	-1.57	0.179	46.16(9.47)	46.88(9.68)	-0.72	0.542

© 2024 Yu W et al. *JAMA Surgery.*

eTable 6. Quality of Life and Functional Outcome of Patients Followed by VPG and VRG Before Surgery, and 6, and 12 Months After Surgery in Per-Protocol Analysis

A. EORTC-QLQ-C30

Variables	Before surgery				6 months after surgery				12 months after surgery			
	mean(SD)		Mean diff.	*P* value	mean(SD)		Mean diff.	*P* value	mean(SD)		Mean diff.	*P* value
	VPG(N=128)	VRG(N=128)			VPG(N=128)	VRG(N=128)			VPG(N=128)	VRG(N=128)		
**Function scales**												
Global health status	76.95(8.95)	78.34(10.45)	-1.39	0.254	56.23(9.42)	57.41(9.75)	-1.18	0.326	64.16(10.00)	64.27(9.49)	-0.12	0.923
Physical functioning	87.52(7.64)	87.00(8.31)	0.52	0.606	70.59(9.78)	69.82(9.53)	0.77	0.522	75.17(8.68)	75.37(9.14)	-0.20	0.861
Emotional functioning	83.70(8.61)	84.97(8.56)	-1.27	0.237	77.54(8.86)	77.13(9.69)	0.41	0.722	85.16(8.46)	83.86(9.04)	1.30	0.234
Cognitive functioning	89.46(6.81)	88.81(6.65)	0.65	0.441	85.05(9.72)	83.35(9.47)	1.70	0.159	86.71(7.40)	87.52(7.40)	-0.80	0.385
Social functioning	87.84(7.70)	86.91(7.70)	0.92	0.339	70.86(8.99)	72.20(10.49)	-1.34	0.275	79.81(8.84)	79.49(8.85)	0.32	0.772
**Symptom scales**												
Fatigue	13.48(8.22)	12.56(7.90)	0.91	0.365	22.77(9.72)	23.80(9.77)	-1.03	0.398	19.05(10.21)	17.94(8.96)	1.11	0.356
Nausea and vomiting	10.56(7.58)	10.35(6.83)	0.21	0.815	17.14(9.34)	19.37(7.73)	-2.23	0.039	14.37(8.61)	14.52(8.39)	-0.16	0.883
Pain	10.73(7.10)	11.29(7.30)	-0.55	0.538	12.68(8.09)	13.84(9.31)	-1.16	0.286	12.20(7.87)	12.04(7.71)	0.16	0.873
Insomnia	14.27(8.67)	15.63(8.70)	-1.36	0.212	25.08(10.39)	25.70(9.39)	-0.63	0.614	19.66(8.69)	19.43(9.10)	0.23	0.839
Appetite loss	11.72(7.13)	11.39(7.38)	0.33	0.718	15.02(8.04)	17.91(8.22)	-2.88	0.005	11.68(7.89)	14.98(7.68)	-3.30	0.001
Constipation	14.12(8.47)	13.34(8.90)	0.77	0.477	17.59(9.43)	18.47(8.78)	-0.88	0.439	15.39(9.02)	15.00(9.21)	0.39	0.732
Diarrhea	12.04(8.15)	12.51(8.39)	-0.47	0.651	23.87(9.90)	24.04(10.66)	-0.17	0.894	18.15(9.25)	18.49(8.75)	-0.34	0.760
Financial difficulties	13.76(7.98)	15.40(9.69)	-1.64	0.141	24.43(10.09)	25.70(9.06)	-1.27	0.292	28.82(10.54)	28.63(10.42)	0.19	0.886

© 2024 Yu W et al. *JAMA Surgery.*

B. EORTC-QLQ-STO22												
Variables	Before surgery				6 months after surgery				12 months after surgery			
	mean(SD)		Mean diff.	*P* value	mean(SD)		Mean diff.	*P* value	mean(SD)		Mean diff.	*P* value
	VPG(N=128)	VRG(N=128)			VPG(N=128)	VRG(N=128)			VPG(N=128)	VRG(N=128)		
Dysphagia	12.01(6.89)	12.41(7.59)	-0.41	0.654	21.90(10.25)	20.00(9.78)	1.90	0.131	16.56(7.93)	15.85(8.98)	0.71	0.503
Pain	10.22(6.93)	10.28(6.86)	-0.06	0.942	16.63(8.87)	16.47(8.23)	0.16	0.884	14.98(8.58)	14.36(7.25)	0.63	0.530
Reflux	11.45(6.99)	11.99(6.76)	-0.55	0.525	15.59(8.75)	18.93(8.31)	-3.34	0.002	12.58(8.55)	15.27(8.20)	-2.70	0.011
Eating	10.59(7.00)	11.68(7.46)	-1.09	0.228	15.54(8.29)	19.43(8.13)	-3.89	<0.001	13.08(9.23)	15.45(8.52)	-2.38	0.033
Anxiety	31.04(9.58)	28.80(11.10)	2.24	0.085	32.55(9.94)	32.03(10.82)	0.52	0.687	25.42(10.21)	27.71(10.11)	-2.29	0.073
Dry mouth	26.71(10.49)	24.64(9.51)	2.07	0.099	26.09(9.80)	26.74(9.01)	-0.65	0.900	20.01(9.46)	21.61(9.44)	-1.60	0.176
Taste	11.04(7.16)	9.84(7.48)	1.20	0.193	14.34(8.59)	13.57(9.05)	0.77	0.484	12.23(7.80)	12.93(8.22)	-0.70	0.488
Body image	15.23(9.06)	16.16(8.03)	-0.92	0.390	22.75(9.76)	24.09(10.09)	-1.34	0.280	18.52(7.67)	17.55(9.37)	0.97	0.366
Hair loss	34.69(10.25)	34.66(9.85)	0.02	0.985	39.54(10.08)	41.10(9.19)	-1.56	0.196	46.19(9.60)	46.90(9.83)	-0.71	0.559

© 2024 Yu W et al. *JAMA Surgery.*

eTable 7. Quality of Life and Functional Outcomes in Patients Receiving Postoperative Adjuvant Chemotherapy Followed by VPG and VRG Before Surgery, and 6, and 12 Months After Surgery in Intention-to-Treat Analysis

A. EORTC-QLQ-C30												
Variables	Before surgery				6 months after surgery				12 months after surgery			
	mean(SD)		Mean diff.	*P* value	mean(SD)		Mean diff.	*P* value	mean(SD)		Mean diff.	*P* value
	VPG(N=18)	VRG(N=14)			VPG(N=18)	VRG(N=14)			VPG(N=18)	VRG(N=14)		
**Function scales**												
Global health status	76.72 (10.75)	78.36 (11.57)	-1.64	0.685	59.39 (9.03)	60.36 (9.60)	-0.97	0.773	62.56(14.37)	66.86 (8.52)	-4.30	0.301
Physical functioning	86.50 (9.26)	87.93 (6.88)	-1.43	0.620	72.72 (12.49)	69.57 (6.69)	3.15	0.369	78.28 (9.17)	76.21 (7.71)	2.07	0.494
Emotional functioning	83.44 (8.33)	85.29 (10.70)	-1.85	0.599	73.11 (6.49)	73.21(11.16)	-0.10	0.977	88.61 (8.35)	79.57(10.75)	9.04	0.016
Cognitive functioning	89.33 (6.76)	89.14 (5.79)	0.19	0.932	82.83 (12.84)	82.93 (9.46)	-0.10	0.980	85.11 (6.79)	85.21 (8.08)	-0.10	0.971
Social functioning	88.61 (8.85)	87.57 (6.86)	1.04	0.711	71.33 (8.86)	74.07 (9.58)	-2.74	0.414	76.11 (7.32)	81.71 (6.88)	-5.60	0.034
**Symptom scales**												
Fatigue	11.72 (7.39)	10.50 (7.99)	1.22	0.662	23.83 (10.64)	29.14 (8.09)	-5.31	0.119	16.78 (8.31)	14.71 (7.09)	2.07	0.454
Nausea and vomiting	11.56 (8.69)	8.71 (7.51)	2.85	0.328	19.22 (10.87)	21.07 (7.96)	-1.85	0.583	14.28 (9.08)	14.14 (8.37)	0.14	0.964
Pain	9.28 (5.92)	13.21 (6.18)	-3.93	0.080	10.78 (7.58)	12.64 (9.60)	-1.86	0.557	14.89 (7.58)	10.71 (7.50)	4.18	0.131
Insomnia	14.78 (6.35)	14.21 (10.02)	0.57	0.854	24.33 (11.60)	27.71 (9.24)	-3.38	0.366	18.78 (9.82)	21.00 (8.53)	-2.22	0.500
Appetite loss	9.78 (5.93)	12.07 (9.29)	-2.29	0.431	10.72 (6.97)	16.29 (7.71)	-5.57	0.044	10.11 (6.43)	13.36 (7.89)	-3.25	0.222
Constipation	13.44 (9.08)	14.29 (8.61)	-0.85	0.789	13.11 (9.26)	16.21 (8.99)	-3.10	0.348	16.00 (8.35)	14.64 (8.17)	1.36	0.647
Diarrhea	10.89 (8.64)	11.14 (8.51)	-0.25	0.935	21.61 (9.94)	24.64 (8.46)	-3.03	0.360	18.78 (6.83)	18.64(10.04)	0.14	0.965
Financial difficulties	13.67 (7.80)	12.36 (10.13)	1.31	0.692	24.89 (11.43)	21.57 (7.72)	3.32	0.336	23.56(11.23)	35.21(10.44)	-11.65	0.005

© 2024 Yu W et al. *JAMA Surgery.*

B. EORTC-QLQ-STO22												
**Variables**	**Before surgery**				**6 months after surgery**				**12 months after surgery**			
	**mean(SD)**		**Mean diff.**	***P* value**	**mean(SD)**		**Mean diff.**	***P* value**	**mean(SD)**		**Mean diff.**	***P* value**
	**VPG(N=18)**	**VRG(N=14)**			**VPG(N=18)**	**VRG(N=14)**			**VPG(N=18)**	**VRG(N=14)**		
Dysphagia	14.00 (8.76)	12.71 (7.69)	1.29	0.661	21.78 (12.71)	26.50 (11.15)	-4.72	0.273	13.17 (7.92)	13.00 (8.16)	0.17	0.953
Pain	10.00 (6.45)	7.64 (5.36)	2.36	0.268	17.39 (8.76)	14.79 (4.77)	2.60	0.293	15.56 (7.90)	15.36 (10.46)	0.20	0.953
Reflux	8.61 (6.41)	10.79 (8.12)	-2.18	0.418	13.11 (8.09)	17.93 (6.11)	-4.82	0.064	8.89 (6.84)	16.79 (8.17)	-7.90	0.007
Eating	10.56 (7.38)	10.57 (7.36)	-0.01	0.997	15.83 (6.53)	19.00 (9.11)	-3.17	0.283	16.06 (11.26)	18.36 (6.92)	-2.30	0.483
Anxiety	36.06 (8.04)	33.50 (8.71)	2.56	0.401	35.56 (10.94)	30.86 (8.77)	4.70	0.188	27.61 (12.93)	24.50 (13.73)	3.11	0.520
Dry mouth	29.06 (8.07)	24.14 (8.44)	4.92	0.107	24.94 (9.36)	31.64 (8.78)	-6.70	0.046	20.22 (11.04)	22.29 (8.95)	-2.07	0.562
Taste	9.50 (8.87)	8.07 (6.18)	1.43	0.595	12.17 (7.11)	14.50 (9.22)	-2.33	0.442	10.78 (8.08)	13.64 (8.12)	-2.86	0.330
Body image	15.39 (9.13)	17.29 (7.26)	-1.90	0.517	25.39 (9.01)	23.93 (13.46)	1.46	0.730	19.33 (5.36)	16.64 (8.27)	2.69	0.303
Hair loss	36.39 (8.08)	32.57 (12.56)	3.82	0.334	38.17 (7.88)	45.57 (9.75)	-7.40	0.029	43.39 (10.64)	47.86 (8.85)	-4.47	0.205

© 2024 Yu W et al. *JAMA Surgery.*

eTable 8. Quality of Life and Functional Outcomes in Patients Not Receiving Postoperative Adjuvant Chemotherapy Followed by VPG and VRG Before Surgery, and 6, and 12 Months After Surgery in Intention-to-Treat Analysis

**A. EORTC-QLQ-C30**												
**Variables**	**Before surgery**				**6 months after surgery**				**12 months after surgery**			
	**mean(SD)**		**Mean diff.**	***P* value**	**mean(SD)**		**Mean diff.**	***P* value**	**mean(SD)**		**Mean diff.**	***P* value**
	**VPG(N=114)**	**VRG(N=118)**			**VPG(N=114)**	**VRG(N=118)**			**VPG(N=114)**	**VRG(N=118)**		
**Function scales**												
Global health status	77.02 (8.52)	78.36 (10.19)	-1.34	0.278	55.73 (9.25)	57.04 (9.58)	-1.31	0.290	64.42 (9.00)	63.97 (9.43)	0.45	0.710
Physical functioning	87.67 (7.26)	86.91 (8.34)	0.76	0.459	70.25 (9.12)	69.86 (9.69)	0.39	0.752	74.72 (8.39)	75.28 (9.17)	-0.56	0.628
Emotional functioning	83.72 (8.55)	84.91 (8.18)	-1.19	0.280	78.22 (8.85)	77.58 (9.28)	0.64	0.591	84.61 (8.23)	84.36 (8.57)	0.25	0.821
Cognitive functioning	89.46 (6.73)	88.75 (6.66)	0.71	0.420	85.39 (8.98)	83.37 (9.35)	2.02	0.095	86.93 (7.38)	87.75 (7.20)	-0.82	0.393
Social functioning	87.69 (7.40)	86.81 (7.69)	0.88	0.375	70.81 (8.89)	71.98(10.43)	-1.17	0.358	80.39 (8.80)	79.19 (8.92)	1.20	0.303
**Symptom scales**												
Fatigue	13.78 (8.20)	12.80 (7.75)	0.98	0.351	22.61 (9.43)	23.13 (9.63)	-0.52	0.678	19.42(10.29)	18.28 (8.96)	1.14	0.370
Nausea and vomiting	10.39 (7.28)	10.56 (6.65)	-0.17	0.853	16.82 (8.93)	19.18 (7.59)	-2.36	0.031	14.39 (8.42)	14.55 (8.29)	-0.16	0.884
Pain	11.00 (7.15)	11.08 (7.29)	-0.08	0.933	12.94 (8.02)	13.97 (9.15)	-1.03	0.362	11.76 (7.74)	12.20 (7.62)	-0.44	0.663
Insomnia	14.18 (8.86)	15.79 (8.43)	-1.61	0.158	25.17 (10.05)	25.44 (9.25)	-0.27	0.832	19.80 (8.39)	19.25 (9.03)	0.55	0.631
Appetite loss	12.01 (7.16)	11.30 (7.04)	0.71	0.447	15.69 (7.88)	18.06 (8.16)	-2.37	0.025	11.90 (7.96)	15.16 (7.53)	-3.26	0.002
Constipation	14.22 (8.27)	13.20 (8.81)	1.02	0.364	18.26 (9.15)	18.71 (8.61)	-0.45	0.700	15.31 (9.01)	15.05 (9.21)	0.26	0.828
Diarrhea	12.25 (7.96)	12.66 (8.26)	-0.41	0.701	24.19 (9.72)	23.98(10.75)	0.21	0.876	18.04 (9.44)	18.49 (8.49)	-0.45	0.703
Financial difficulties	13.75 (7.92)	15.75 (9.45)	-2.00	0.082	24.37 (9.75)	26.15 (8.96)	-1.78	0.149	29.63(10.05)	27.82(10.00)	1.81	0.171

© 2024 Yu W et al. *JAMA Surgery.*

B. EORTC-QLQ-STO22

Variables	Before surgery				6 months after surgery				12 months after surgery			
	mean(SD)		Mean diff.	*P* value	mean(SD)		Mean diff.	*P* value	mean(SD)		Mean diff.	*P* value
	VPG(N=114)	VRG(N=118)			VPG(N=114)	VRG(N=118)			VPG(N=114)	VRG(N=118)		
Dysphagia	11.73 (6.43)	12.42 (7.49)	-0.69	0.452	21.91 (9.69)	19.24 (9.19)	2.67	0.032	17.11 (7.72)	16.18 (8.90)	0.93	0.396
Pain	10.26 (6.93)	10.58 (6.86)	-0.32	0.724	16.49 (8.79)	16.69 (8.42)	-0.20	0.860	14.88 (8.60)	14.25 (6.70)	0.63	0.535
Reflux	11.89 (6.90)	12.13 (6.50)	-0.24	0.785	15.96 (8.68)	19.02 (8.42)	-3.06	0.007	13.18 (8.53)	15.08 (8.08)	-1.90	0.083
Eating	10.61 (6.87)	11.79 (7.37)	-1.18	0.208	15.46 (8.42)	19.47 (7.91)	-4.01	＜0.001	12.60 (8.66)	15.10 (8.51)	-2.50	0.028
Anxiety	30.23 (9.43)	28.22 (11.07)	2.01	0.137	32.07 (9.56)	32.15 (10.90)	-0.08	0.953	25.08 (9.55)	28.07 (9.42)	-2.99	0.017
Dry mouth	26.29 (10.63)	24.69 (9.51)	1.60	0.229	26.24 (9.73)	26.16 (8.74)	0.08	0.948	19.98 (9.07)	21.56 (9.38)	-1.58	0.193
Taste	11.28 (6.75)	10.06 (7.50)	1.22	0.194	14.67 (8.64)	13.47 (8.91)	1.20	0.299	12.45 (7.63)	12.82 (8.13)	-0.37	0.721
Body image	15.21 (8.93)	16.02 (8.00)	-0.81	0.468	22.32 (9.68)	24.11 (9.52)	-1.79	0.157	18.38 (7.87)	17.67 (9.37)	0.71	0.532
Hair loss	34.39 (10.39)	34.89 (9.36)	-0.50	0.701	39.72 (10.24)	40.55 (8.85)	-0.83	0.510	46.60 (9.25)	46.76 (9.80)	-0.16	0.898

© 2024 Yu W et al. *JAMA Surgery.*

eFigure 1. Diagram For Perigastric Vagus Nerve and the Preservation of Vagus Nerve During Distal Gastrectomy

A.

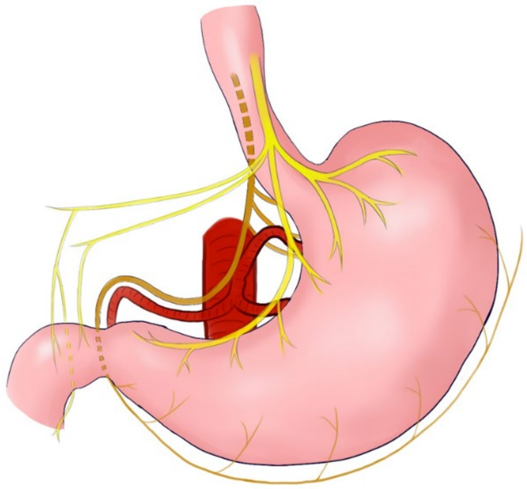

B.

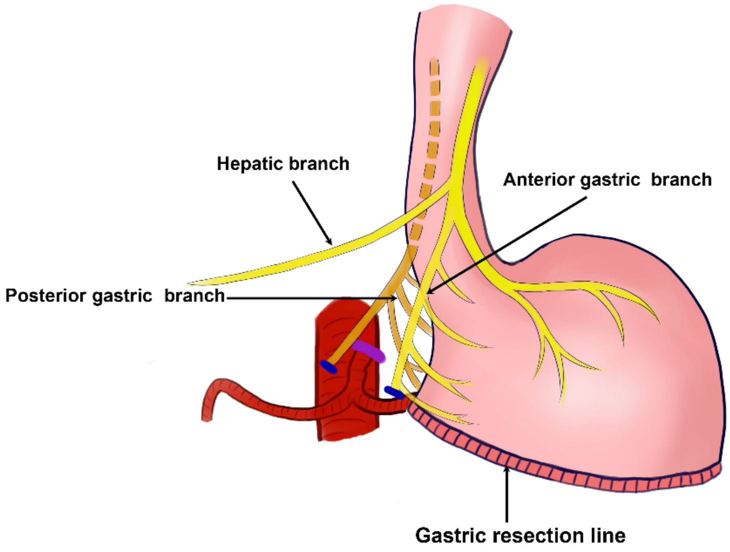

© 2024 Yu W et al. *JAMA Surgery.*

eFigure 2. The Preservation of the Vagus Nerve During Distal Gastrectomy

A.

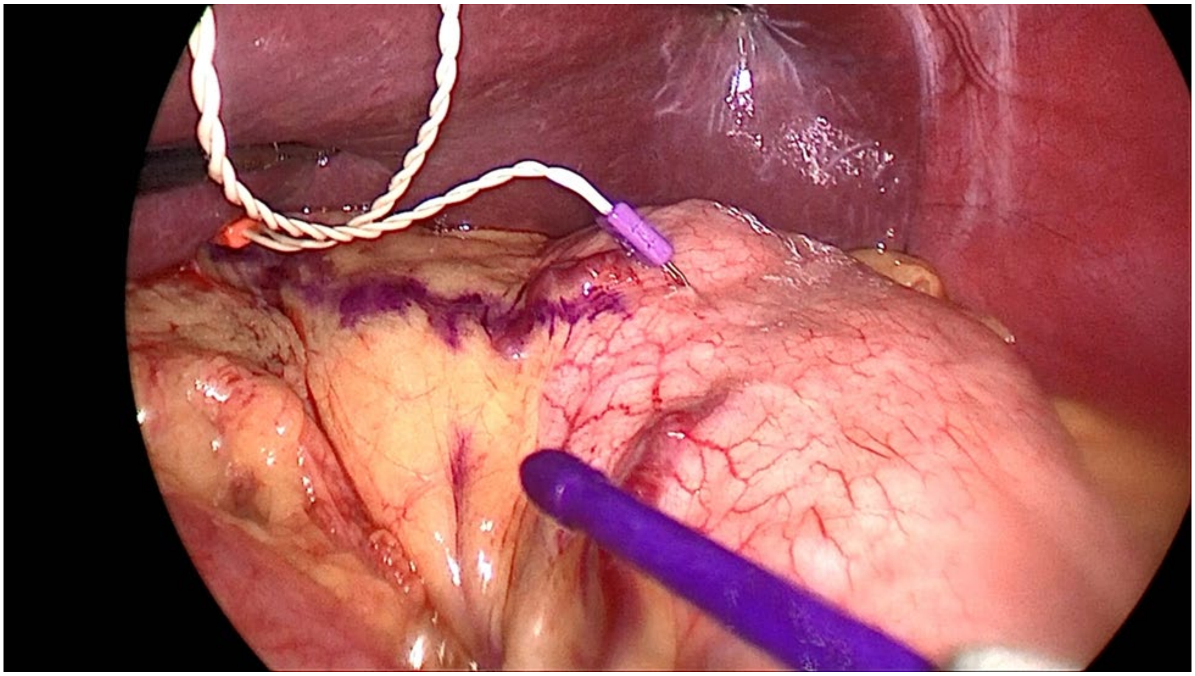

B.

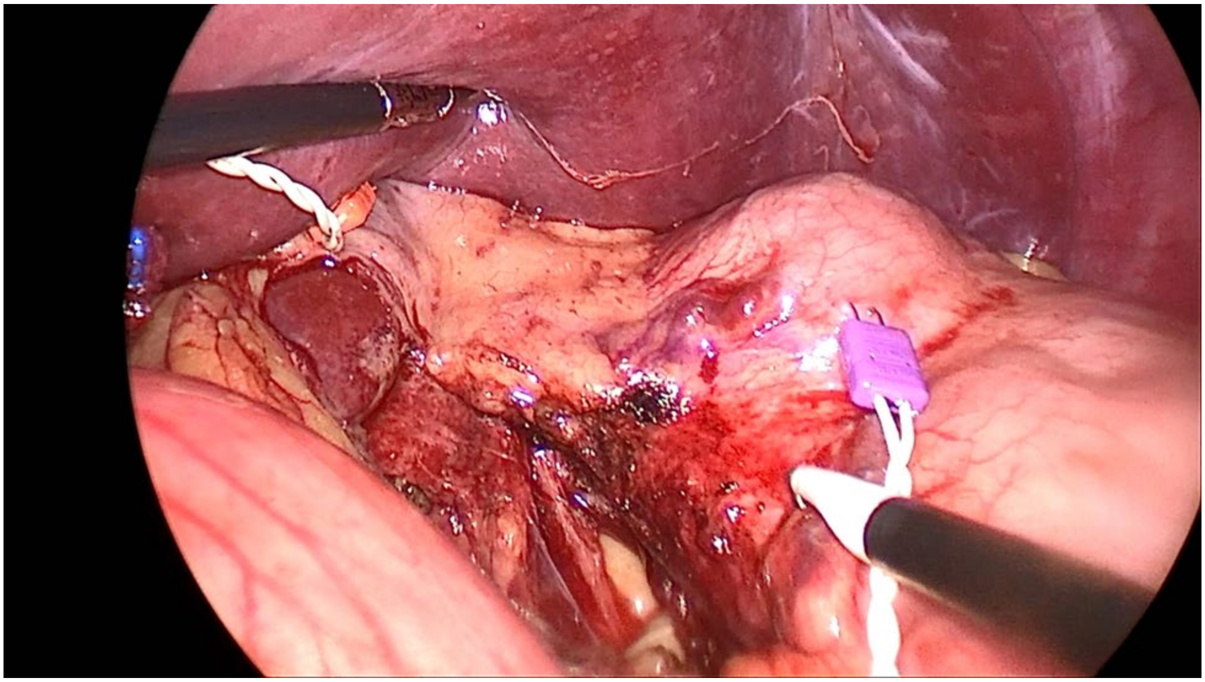

© 2024 Yu W et al. *JAMA Surgery.*

eFigure 3. The Functional Scales of the EORTC QLQ-C30

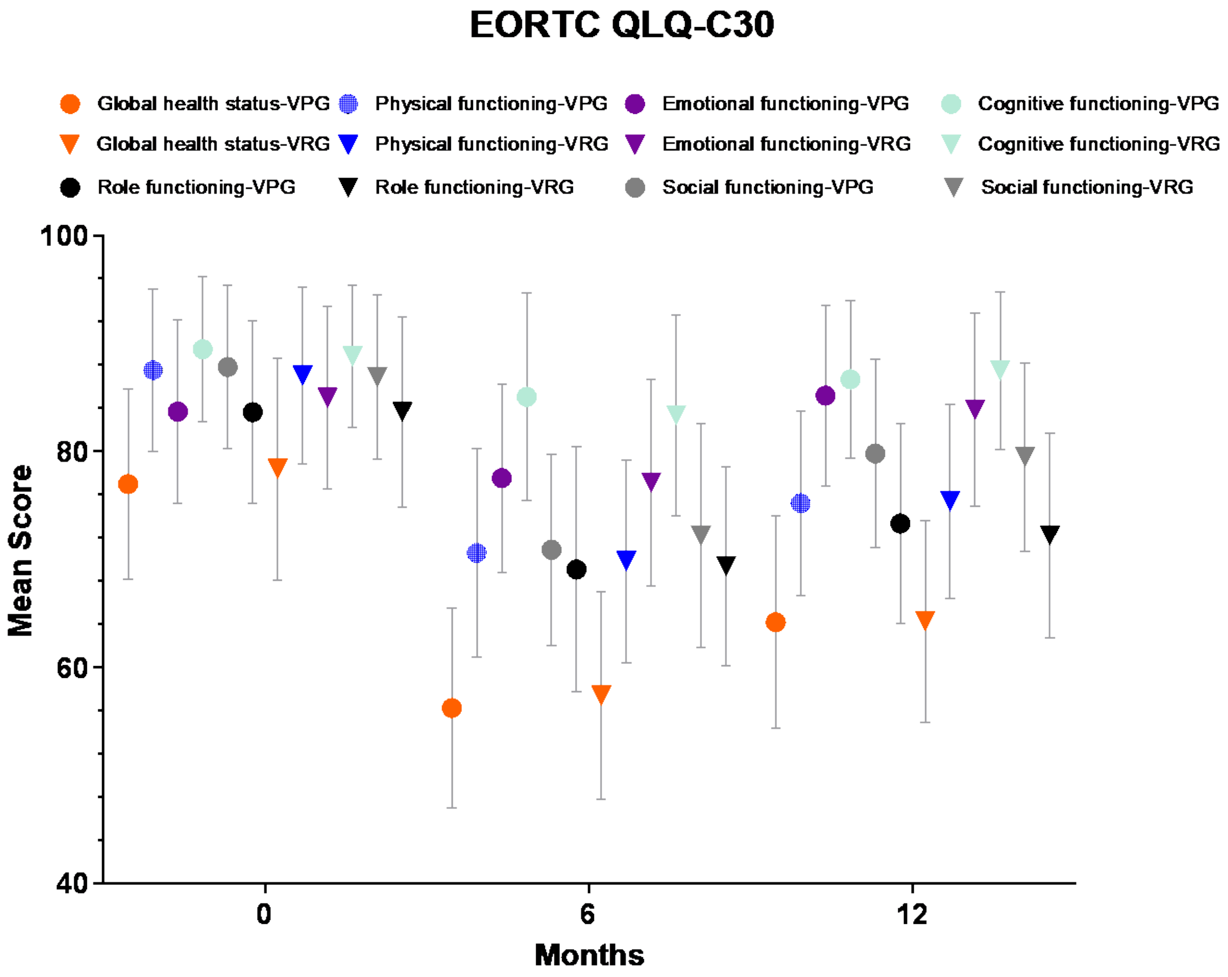

© 2024 Yu W et al. *JAMA Surgery.*

eFigure 4. The Fatigue, Pain, Dysponea, and Insomnia Scales of the EORTC QLQ-C30

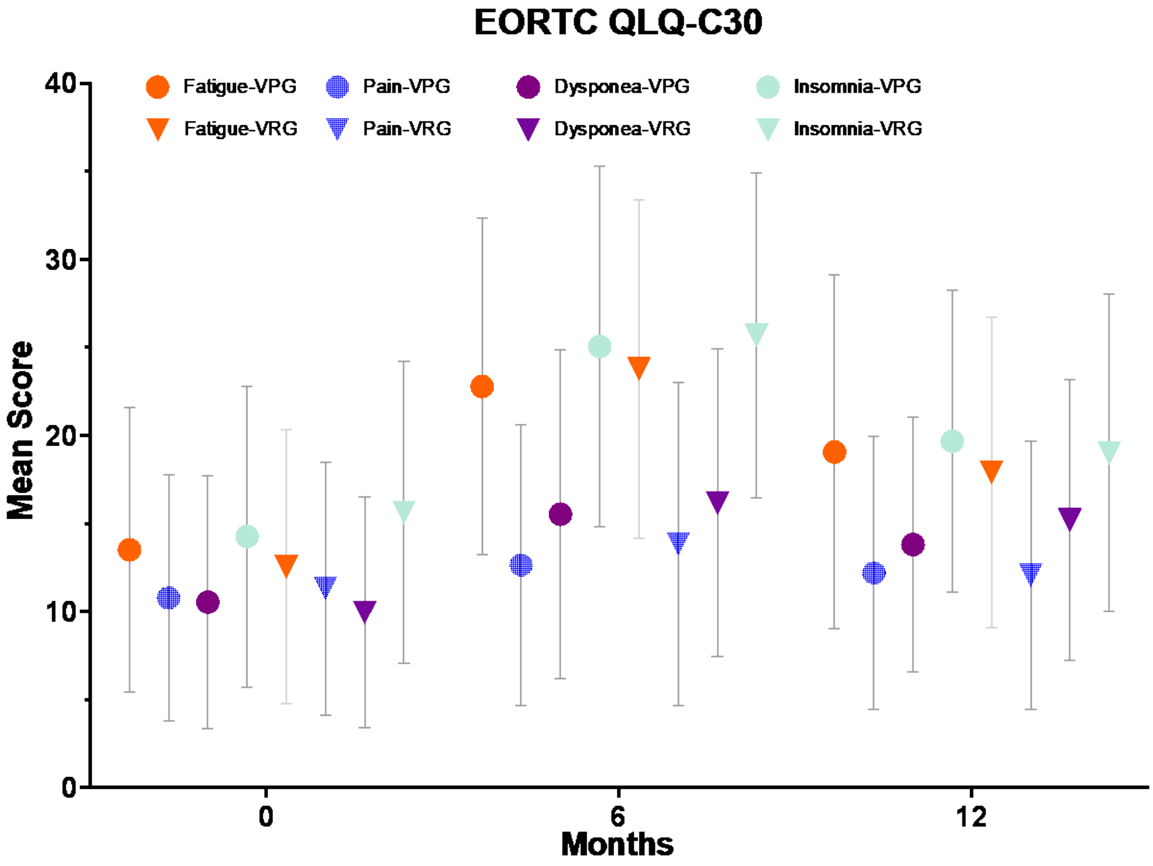

© 2024 Yu W et al. *JAMA Surgery.*

eFigure 5. The Constipation, Diarrhea, and Financial Difficulties Scales of the EORTC QLQ-C30

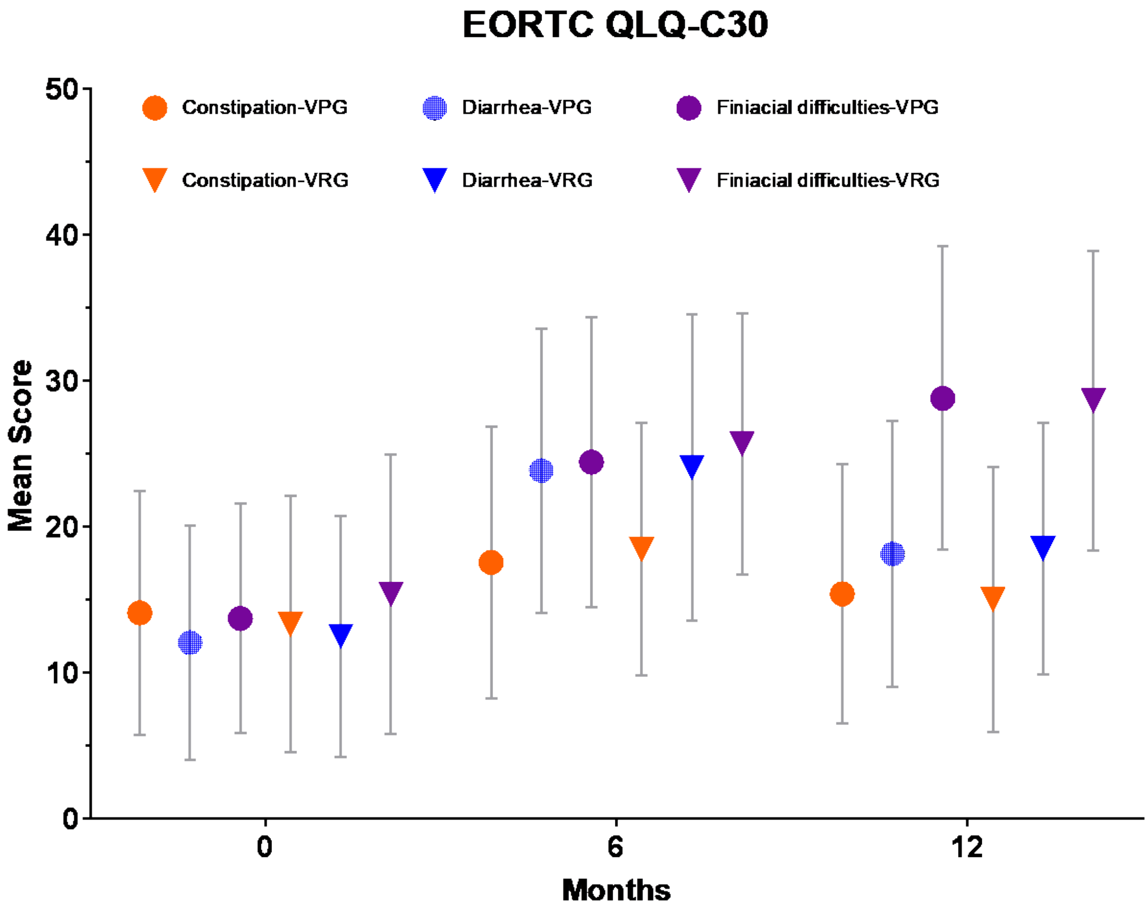

© 2024 Yu W et al. *JAMA Surgery.*

eFigure 6. The Dysphagia, Pain, and Anxiety Scales of the EORTC QLQ-STO22

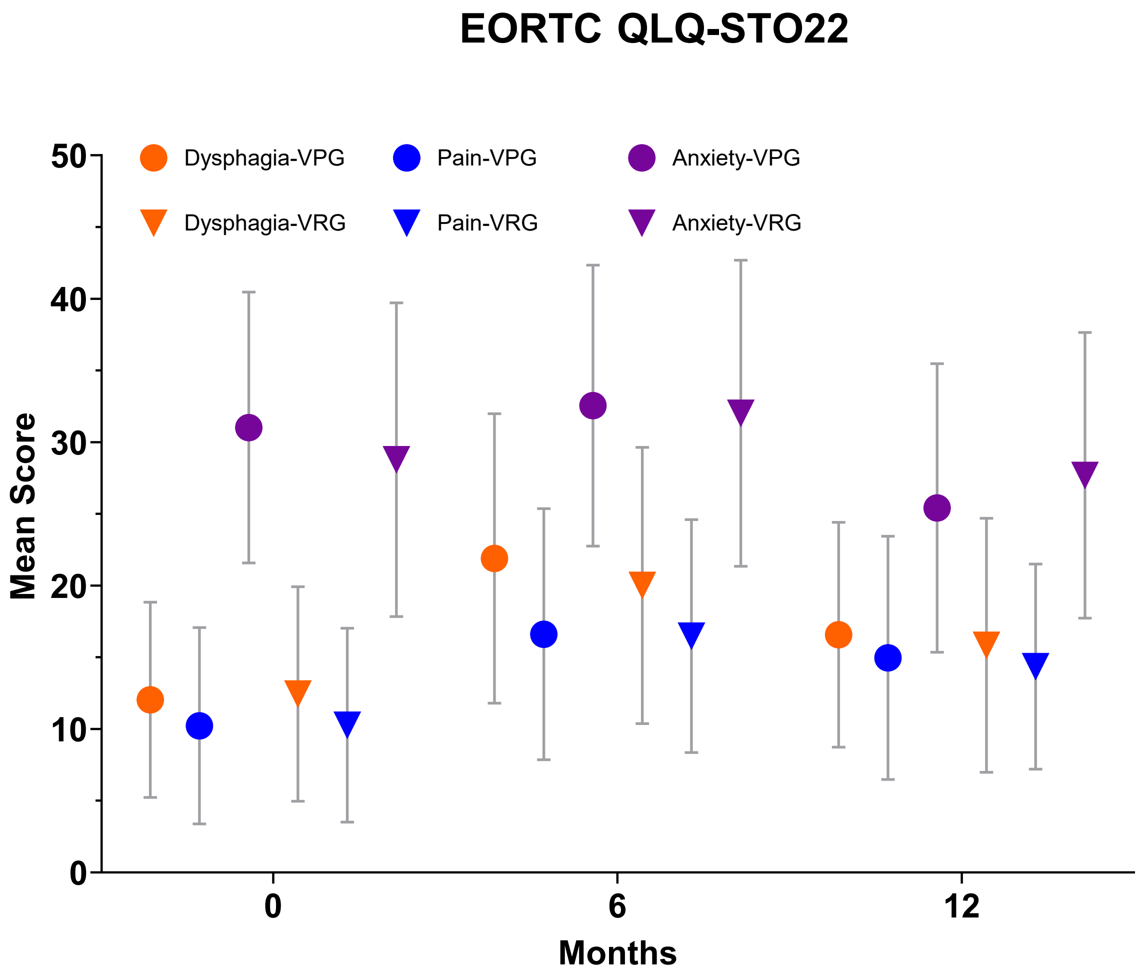

© 2024 Yu W et al. *JAMA Surgery.*

eFigure 7. The Dry Mouth, Taste, Body Image, and Hair Loss Scales of the EORTC QLQ-STO22

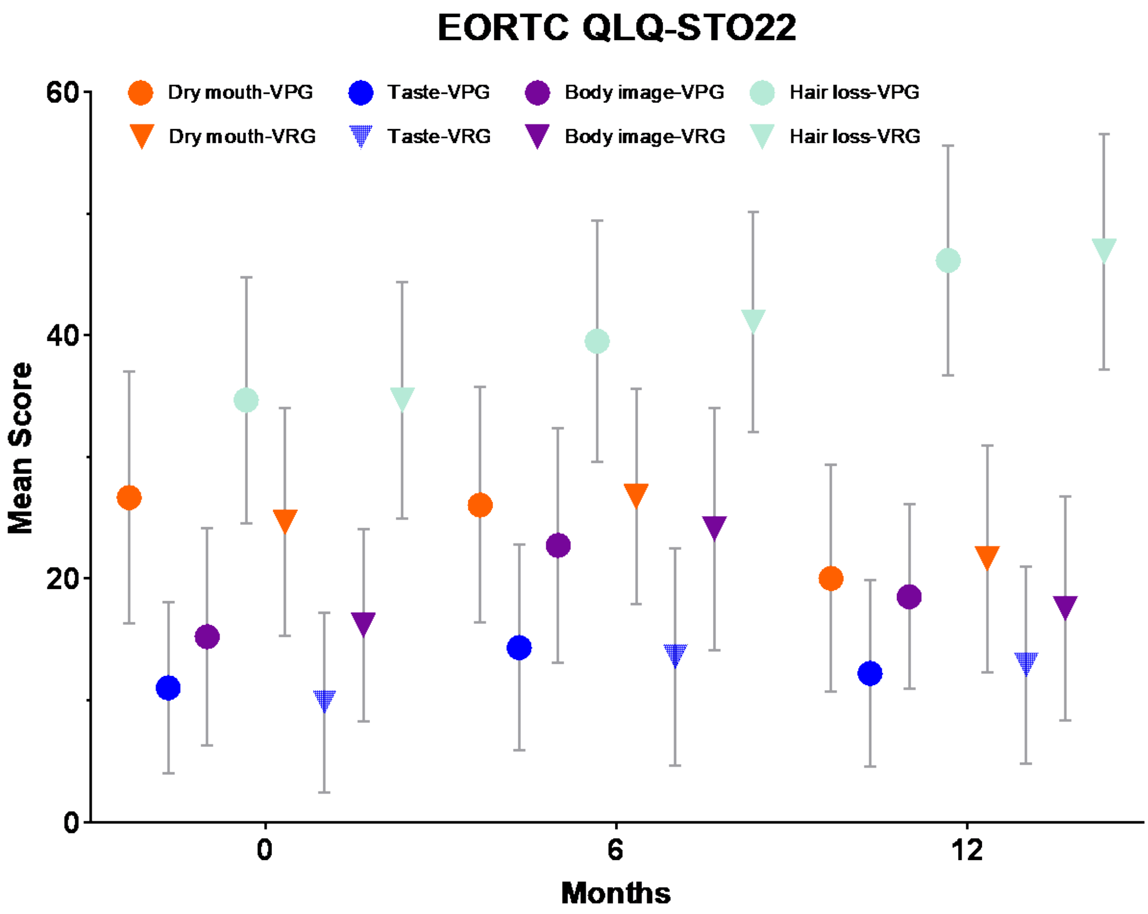

© 2024 Yu W et al. *JAMA Surgery.*
