# Supplementary material for: Vagus Nerve Preservation for Early Distal Gastric Cancer With Monitoring and Indocyanine Green Labeling: A Randomized Clinical Trial
Source: JAMA Surg. 2024 Nov 13:e245077. Online ahead of print. doi: 10.1001/jamasurg.2024.5077 (PMC11561724; doi:10.1001/jamasurg.2024.5077)
Supplement: Supplement 3. — Data Sharing Statement [file jamasurg-e245077-s003.pdf]

Data Sharing Statement

Yan. Vagus Nerve Preservation for Early Distal Gastric Cancer With Monitoring and Indocyanine Green Labeling. *JAMA Surg*. Published November 13, 2024. doi:10.1001/jamasurg.2024.5077

Data
Additional Information: Chictr.org.cn https://www.chictr.org.cn/showprojEN.html?proj=166485 ChiCTR2200059489
Data available: Yes
Data types: Deidentified participant data
How to access data: How to access data: wenbin_yu2003@163.com When available: With publication Supporting Documents Document types: Other (please specify) Additional Information: Trial protocol How to access documents: wenbin_yu2003@163.com When available: With publication Additional Information Who can access the data: Wenbin Yu Types of analyses: for a specified purpose Mechanisms of data availability: after approval of a proposal
When available: With publication

Supporting Documents
Document types: Informed consent form
How to access documents: Who can access the data: Wenbin Yu
When available: With publication

Additional Information
Who can access the data: Who can access the data: Wenbin Yu
Types of analyses: all the analysis data
Mechanisms of data availability: Mechanisms of data availability: after approval of a proposal
